# Assembly of eukaryotic algal chromosomes in yeast

**DOI:** 10.1186/1754-1611-7-30

**Published:** 2013-12-10

**Authors:** Bogumil J Karas, Bhuvan Molparia, Jelena Jablanovic, Wolfgang J Hermann, Ying-Chi Lin, Christopher L Dupont, Christian Tagwerker, Isaac T Yonemoto, Vladimir N Noskov, Ray-Yuan Chuang, Andrew E Allen, John I Glass, Clyde A Hutchison, Hamilton O Smith, J Craig Venter, Philip D Weyman

**Affiliations:** 1 Department of Synthetic Biology and Bioenergy, J. Craig Venter Institute, 10355 Science Center Dr., San Diego, CA 92121, USA; 2 Department of Microbial and Environmental Genomics, J. Craig Venter Institute, 10355 Science Center Dr., San Diego, CA 92121, USA; 3 Department of Synthetic Biology and Bioenergy, J. Craig Venter Institute, 9704 Medical Center Dr., Rockville, MD 20850, USA

**Keywords:** Eukaryote, Chromosome, TAR cloning, *Saccharomyces cerevisiae*, Autonomously replicating sequence (ARS), *Phaeodactylum tricornutum*

## Abstract

**Background:**

Synthetic genomic approaches offer unique opportunities to use powerful yeast and *Escherichia coli* genetic systems to assemble and modify chromosome-sized molecules before returning the modified DNA to the target host. For example, the entire 1 Mb *Mycoplasma mycoides* chromosome can be stably maintained and manipulated in yeast before being transplanted back into recipient cells. We have previously demonstrated that cloning in yeast of large (> ~ 150 kb), high G + C (55%) prokaryotic DNA fragments was improved by addition of yeast replication origins every ~100 kb. Conversely, low G + C DNA is stable (up to at least 1.8 Mb) without adding supplemental yeast origins. It has not been previously tested whether addition of yeast replication origins similarly improves the yeast-based cloning of large (*>*150 kb) eukaryotic DNA with moderate G + C content. The model diatom *Phaeodactylum tricornutum* has an average G + C content of 48% and a 27.4 Mb genome sequence that has been assembled into chromosome-sized scaffolds making it an ideal test case for assembly and maintenance of eukaryotic chromosomes in yeast.

**Results:**

We present a modified chromosome assembly technique in which eukaryotic chromosomes as large as ~500 kb can be assembled from cloned ~100 kb fragments. We used this technique to clone fragments spanning *P. tricornutum* chromosomes 25 and 26 and to assemble these fragments into single, chromosome-sized molecules. We found that addition of yeast replication origins improved the cloning, assembly, and maintenance of the large chromosomes in yeast. Furthermore, purification of the fragments to be assembled by electroelution greatly increased assembly efficiency.

**Conclusions:**

Entire eukaryotic chromosomes can be successfully cloned, maintained, and manipulated in yeast. These results highlight the improvement in assembly and maintenance afforded by including yeast replication origins in eukaryotic DNA with moderate G + C content (48%). They also highlight the increased efficiency of assembly that can be achieved by purifying fragments before assembly.

## Background

Synthetic biology can be described as the iterative process of modeling biological phenomena, building genetic devices to implement these models, and evaluating device performance. This discipline practiced at the scale of entire chromosomes can be defined as synthetic genomics. Synthetic genomic approaches focus on understanding function and regulation of multi-gene pathways, function of chromosome architecture, and strategies for multi-locus engineering. Several recent examples of synthetic genomic approaches include the creation of a bacterial cell from a chemically-synthesized genome [1], the cloning and manipulation of entire bacterial chromosomes in *Bacillus subtilis*[2,3], and efforts to synthesize and shuffle yeast chromosomes [4-6].

We have previously used the synthetic genomic approach with prokaryotic genomes which are typically small, circular, and contained on a single molecule. These qualities allow for efficient maintenance and manipulation of the chromosome in yeast [7-10]. Once maintained in yeast, chromosomes can be efficiently manipulated before reintroduction into the host. Expanding synthetic genomic techniques to other bacteria is an active research area, but may also be useful for eukaryotic organisms. Chromosomes from eukaryotic organelles have been synthesized or assembled in yeast including the mitochondrion [11] and the chloroplast [12], but to our knowledge, no entire eukaryotic nuclear chromosome has been cloned in yeast.

Large regions of eukaryotic chromosomes have been cloned in yeast. Human (41% G + C) and mouse (42% G + C) YAC libraries have been made with average insert sizes of 620 kb and 700 kb, respectively [13], and human YAC clones greater than 1 Mb have been reported [14,15]. Small DNA fragments from diverse eukaryotes can function as yeast replication origins including *Neurospora crassa* (49% G + C), *Dictyostelium discoideum* (22% G + C), *Caenorhabditis elegans* (35% G + C), *Drosophila melanogaster* (42% G + C), and *Zea mays* (47% G + C) [16]. Addition of yeast origins of replication was previously shown to improve maintenance of large regions of prokaryotic DNA, especially when the sequence has a higher average G + C content than yeast [17]. DNA with low G + C content will have a greater chance of having A + T-rich sites that function as yeast replication origins while DNA of higher average G + C content will have statistically fewer of these sites (Table 1). Given that some sequences from diverse eukaryotes can function as yeast replication origins, we sought to test whether adding additional yeast replication origins could improve maintenance of entire eukaryotic chromosomes from non-fungal species.

**Table 1:** 11-base yeast ARS consensus sequence

**Organism**	**Chromosome size (kb)**	**11 base consensus count (WTTTAYRTTTW)**	**Average consensus per base pair**	**Longest region without consensus sequence (kb)**
*Mycoplasma mycoides*	1084	303	0.28	22.5
*Acholeplasma laidlawii*	1498	179	0.12	65.2
*Prochlorococcus MED4*	1658	328	0.20	34.5
*P. tricornutum* chromosome 25	497	12	0.02	235
*P. tricornutum* chromosome 26	441	8	0.02	188

To demonstrate the ability of yeast to maintain entire eukaryotic chromosomes, we investigated the yeast-based assembly of chromosomes from the diatom *Phaeodactylum tricornutum*. This marine diatom has a total genome size of 27.4 Mb and a G + C content of 48%. After the completely closed genomes of *Ostreococcus lucimarinus*[18] and *Cyanidioschyzon merolae*[19], *P. tricornutum* has one of the best-assembled algal genomes with 33 chromosome-sized scaffolds including 12 with telomere-to-telomere coverage [20]. Additionally, some of the telomere-to-telomere scaffolds are relatively small (<600-kb), making them good targets for developing synthetic genomic approaches. Experiments with the *P. tricornutum* system benefit from efficient transformation methods [21] and a long history of physiological research [22]. Because *P. tricornutum* is an emerging model genetic system for marine microalgal biology and biotechnology [23], we chose it to develop the first stage of a synthetic genomic approach: cloning, maintaining, and manipulating chromosomes in yeast. In this paper, we present data on the prospects and limitations associated with assembling, maintaining and manipulating *P. tricornutum* chromosomes in yeast and demonstrate improvements on the yeast assembly process including gel purification by electroelution as a powerful way to concentrate large DNA fragments for assembly.

## Results

The 27.4 Mb *P. tricornutum* nuclear genome currently exists as 33 individual scaffolds. These scaffolds are described as chromosomes in reports on the genome sequence [20], although ongoing work to completely close the genome sequence may collapse some of these scaffolds without telomere-to-telomere coverage into single chromosomes. To be consistent with published work describing the genome, we refer to these scaffolds as “chromosomes”. We chose *P. tricornutum* chromosomes 25 and 26 (lengths of 497 kb and 441 kb, respectively) as targets to assemble entire eukaryotic chromosomes in yeast because they are the smallest chromosomes for which telomere-to-telomere assembly exists. Chromosomes 25 and 26 each have a single remaining sequencing gap predicted to be 3.7 kb and 17 kb on chromosomes 25 and 26, respectively.

### Cloning fragments spanning chromosomes

To clone an entire eukaryotic chromosome in yeast, we used an assembly-based strategy in which the chromosome was first cloned as smaller fragments in yeast and then assembled into the full-length molecule. Previous results cloning large DNA fragments from bacteria with moderately high G + C content (55%) indicated that fragments greater than *ca*. 150 kb were optimally maintained after addition of a yeast replication origin sequence [17]. With the large bacterial fragments, we demonstrated that adding additional yeast replication origins to the DNA allowed for stable maintenance of a *ca*. 450-kb fragment in yeast. Eukaryotic DNA fragments from sources such as human (41% G + C) and mouse (42% G + C) have been cloned in YACs that exceed 1 Mb, and we sought to test whether *P. tricornutum* DNA fragments (48% G + C) larger than *ca.* 150 kb could be replicated efficiently in yeast because of the higher G + C content.

To test whether additional yeast replication origins would improve the maintenance of larger *P. tricornutum* DNA, we first cloned chromosomes 25 and 26 each as five smaller *ca*. 100-kb fragments. Breaking the chromosome into smaller pieces allowed us to test for toxicity of any genes that may happen to be expressed in yeast and allowed us to further modify the pieces to test whether adding additional yeast origins of replication improved assembly efficiency in yeast.

We cloned the five, large, overlapping fragments that compose a chromosome using a yeast-based method called Transformation-Associated Recombination (TAR) cloning [24-26]. In TAR cloning, a yeast vector containing sequence overlaps with a specific target sequence is co-transformed with genomic DNA containing the target sequence. Yeast performs specific recombination events to recombine the target sequence into the plasmid. The efficiency of this process is greatly improved if the target sequence is linearized near at least one of the sites of recombination. We took advantage of rare-cutting restriction sites present in the *P. tricornutum* chromosomes to design our cloning strategy which would allow us to create double-stranded breaks near at least one of the homologous recombination sites. Digestion sites were selected such that each of the five chromosomal fragments overlapped by 10–40 kb (Table 2, Additional file 1: Figure S1). To clone each fragment, *P. tricornutum* DNA was prepared in agarose plugs to prevent unwanted shearing, and plugs were digested with the appropriate enzyme(s) to yield long DNA fragments (Additional file 1: Figure S1). To clone the *P. tricornutum* chromosome fragments, we designed vectors which had two 200-bp homology regions specific for each fragment. Cloned fragments were selected by the *HIS3* marker on the vector, and, because our vector design required two recombination events resulting in elimination of the *URA3* marker from the vector, empty vector molecules could be eliminated by selecting for the absence of the *URA3* maker using 5-fluoroorotic acid (5FOA, see Methods) [27].

**Table 2:** ***P. tricornutum *** **fragments used for assembly of chromosomes 25 and 26**

**Fragment #**	**Fragment size (kb) (final sizes with KmArs (frags 2,3,4) and Ura (frag 3))**	**Restriction sites used to cut genomic DNA for TAR cloning**	**Release sites (5’ and 3’)**	**5’ overlap (kb)**	**3’ overlap (kb)**
25-1	105.5	NotI (3’ only)	I-CeuI + I-SceI	0.6	35.4
25-2	126.9	FseI (3’ only)	I-CeuI + I-SceI	35.4	9.6
25-3	122.3	SrfI	I-CeuI + I-SceI	9.6	27.8
25-4	125.2	FseI (5’ only)	I-CeuI + I-SceI	27.8	32.8
25-5	127.5	PmeI (5’ only)	I-CeuI + I-SceI	32.8	0.6
26-1	76.6	AscI (3’ only)	I-CeuI + I-SceI	0.5	26.1
26-2	142.6	SwaI	I-CeuI + I-SceI	26.1	39.5
26-3	129.3	NotI + AscI	I-CeuI + I-SceI	39.5	25.1
26-4	116.6	AsiSI	I-CeuI + I-SceI	25.1	12.1
26-5	83.1	AbsI (5’ only)	I-CeuI + I-SceI	12.1	0.5

**Figure 1:**
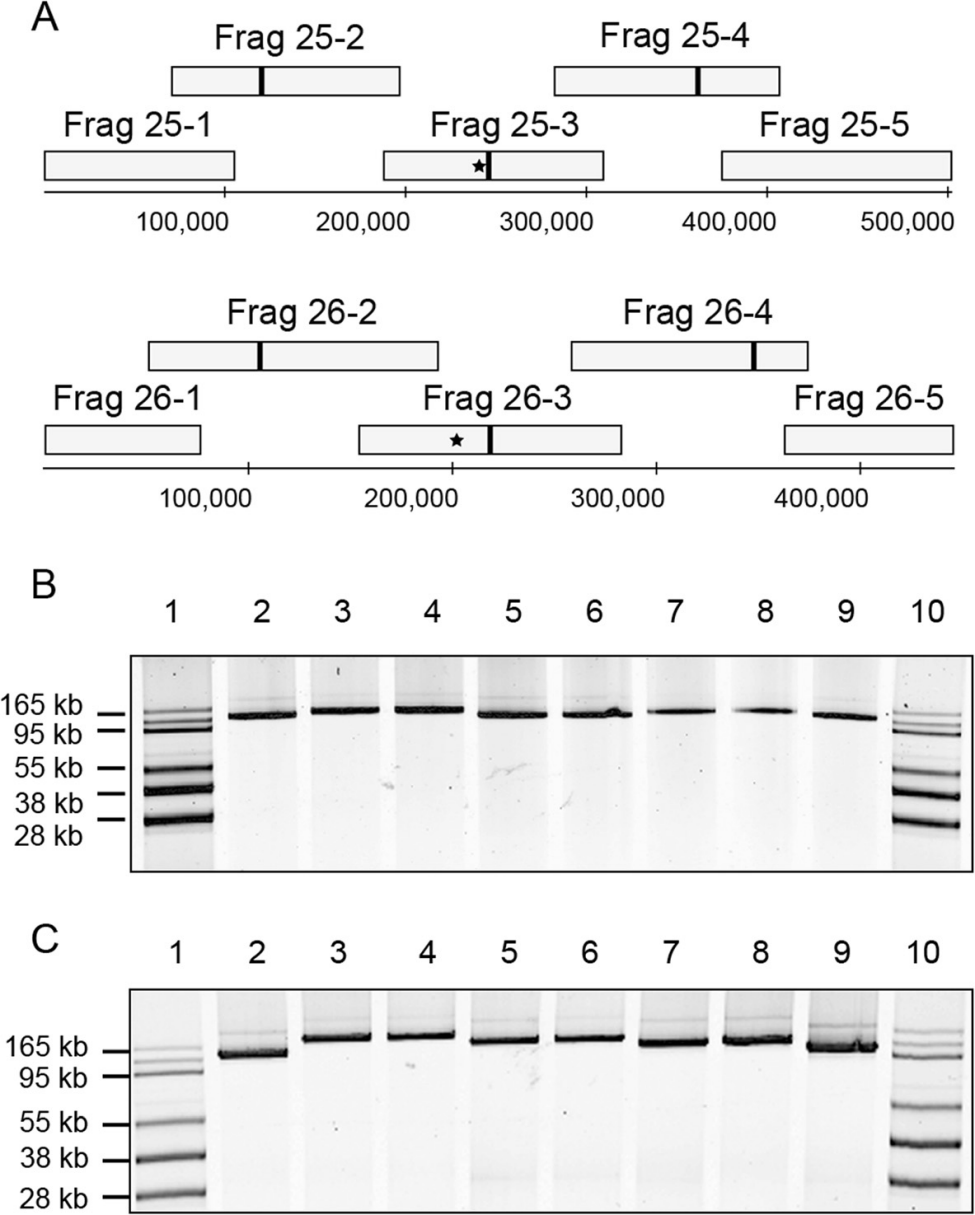
**Cloning of *P. tricornutum *chromosomes as five overlapping fragments. A.** Chromosomes 25 and 26 were each cloned as five overlapping fragments in yeast using a BAC-YAC vector. Fragments were further modified by addition of the *URA3* selectable marker in fragment 3 (asterisk) and the addition of a yeast replication origin (ARS) in fragments 2, 3, and 4 (vertical bar). **B** and **C.** The cloned fragments were purified from yeast, transformed into *E. coli*, and were purified and separated on agarose gels as supercoiled plasmids. Chromosome 25 **(B)** and 26 **(C)** fragments were each in the following order: BAC-Tracker supercoiled DNA ladder (Epicentre, lane 1), Fragment 1 (lane 2), Fragment 2 (larger version ~ 170 kb, lane 3), Fragment 2-ARS (lane 4), Fragment 3-URA (lane 5), Fragment 3-URA-ARS (lane 6), Fragment 4 (lane 7), Fragment 4-ARS (lane 8), Fragment 5 (lane 9), BAC-Tracker ladder (lane 10).

Resulting yeast colonies were screened by PCR using primers designed to amplify specific, small, unique regions (less than 1 kb) located approximately in the middle of each fragment. Clones verified as correct by PCR were purified from yeast and transformed into *E. coli* to obtain higher plasmid yields. The large, cloned, ~100 kb fragments were then separated as supercoiled plasmids on an agarose gel to estimate their size. All fragments from chromosome 25 yielded bands of the expected size (Figure 1 and Table 2). Fragments from chromosome 26 were also of the expected size with the exception of fragments 26–1 and 26–2. For fragment 26–1, we expected a fragment of size 88 kb (including vector sequence) but obtained fragments of around 130 kb (Figure 1). For fragment 26–2, we expected a fragment at around 152 kb (including vector sequence), but we obtained plasmids of two different sizes: one was too small at around 100 kb and one that appeared too large at around 170 kb. Because we worried that the smaller-sized plasmids contained deletions, we chose the larger plasmid (see discussion for further explanation) to proceed with the assembly of chromosome 26.

After all fragments had been cloned in yeast and moved to *E. coli*, a single clone from each of the five fragments was chosen to move forward for assembly of the entire chromosome (Figure 1). To facilitate chromosome assembly and selection of correctly-assembled molecules in yeast, fragment 3 from each of chromosomes 25 and 26 was modified in yeast with an additional uracil selectable marker (*URA3*, Table 3). Each of fragments 2, 3, and 4 from chromosomes 25 and 26 was modified by addition of a yeast replication origin (also called an autonomously replicating sequence or ARS, Table 3). After modifications, plasmids were extracted and separated by agarose gel electrophoresis to verify that the correct plasmid size was maintained (Figure 1).

**Table 3:** **Sites of insertion of yeast replication origins (KmR-ARS) and yeast selectable markers (URA) in cloned *P. tricornutum *** **fragments**

**Fragment**	**KmR-ARS insertion position (nt from beginning of chromosome sequence at 5’ site of insertion)**	**URA insertion position (nt from beginning of chromosome sequence at 5’ site of insertion)**
25-2	120000	NA
25-3	241000	239990
25-4	357000	NA
26-2	115973	NA
26-3	230089	213353
26-4	358818	NA

To further validate the identity of the cloned fragments, we tested the plasmids containing the large *P. tricornutum* fragments using a higher resolution multiplex PCR. The multiplex primer set was designed to amplify a fragment every ~20 kb. All fragments in chromosome 25 had the expected high resolution multiplex PCR pattern with the exception of 25–3 that lacked one band where the primer binding site was interrupted by the *URA3* insertion (Additional file 1: Figure S2) and a faint band present for 25–2 (MPX2, Additional file 1: Figure S2) that was likely the result of non-specific primer binding (see Additional file 1: Figure S2 for additional explanation). High resolution multiplex PCR was also performed after modification with the ARS regions (Additional file 1: Figure S2). Modification with the ARS regions did not alter the multiplex PCR pattern indicating that all regions of the chromosome were still present after modification.

For chromosome 26, fragments 26–1, 26–3, 26–4, and 26–5 had the expected high resolution multiplex bands PCR (Additional file 1: Figure S1). The larger than expected size of 26–1 may be explained by the sequencing gap found within this fragment. High resolution multiplex PCR with several larger 26–2 clones indicated that they were each lacking one of the multiplex bands near the 5’ end of the fragment (Additional file 1: Figure S1). We continued with these fragments because the multiplex PCR indicated that there was still sequence overlap between fragments 26–1 and 26–2. Of all the fragments cloned, 26–1 and 26–2 were the only fragments that yielded a discrepancy in either size or DNA content from what was predicted by the published genome sequence, and we discuss possibilities for these observed differences at the end of the paper.

Our cloning strategy involved capturing sequence immediately adjacent to the telomeric repeats found at the ends of fragments 25–1, 25–5, 26–1, and 26–5. To verify that the regions immediately inside of the telomeric regions were cloned, we amplified the vector-insert junctions of the 5’ region of fragments 25–1 and 26–1 and the 3’ region of fragments 25–5 and 26–5. When these amplicons were sequenced, the results confirmed that the junction was seamless between the subtelomeric region and the vector, and all telomeric repeats had been removed in the TAR-cloning process.

### Assembly of fragments into chromosomes

To assemble whole chromosomes in yeast, plasmids carrying the ~100 kb *P. tricornutum* fragments (with or without added ARS insertion) were purified from *E. coli* and the cloned fragments were released from the cloning vector by restriction enzyme digestion. After digestion, *P. tricornutum* DNA fragments were separated from the vector sequence by agarose gel electrophoresis, and *P. tricornutum* fragments were electroeluted from the agarose gel. The five *P. tricornutum* fragments spanning an entire chromosome were then assembled into a new vector in yeast using spheroplast transformation in one of two experimental configurations. In the first, the five fragments lacked added yeast origins while in the second, fragments 2, 3, and 4 each contained an additional yeast origin. In both configurations, fragment 3 contained an additional *URA3* gene to assist in selection of correct assemblies. Selectable markers on both the vector and fragment 3 complemented tryptophan and uracil auxotrophies, respectively, and improved selection for complete assemblies.

**Figure 2:**
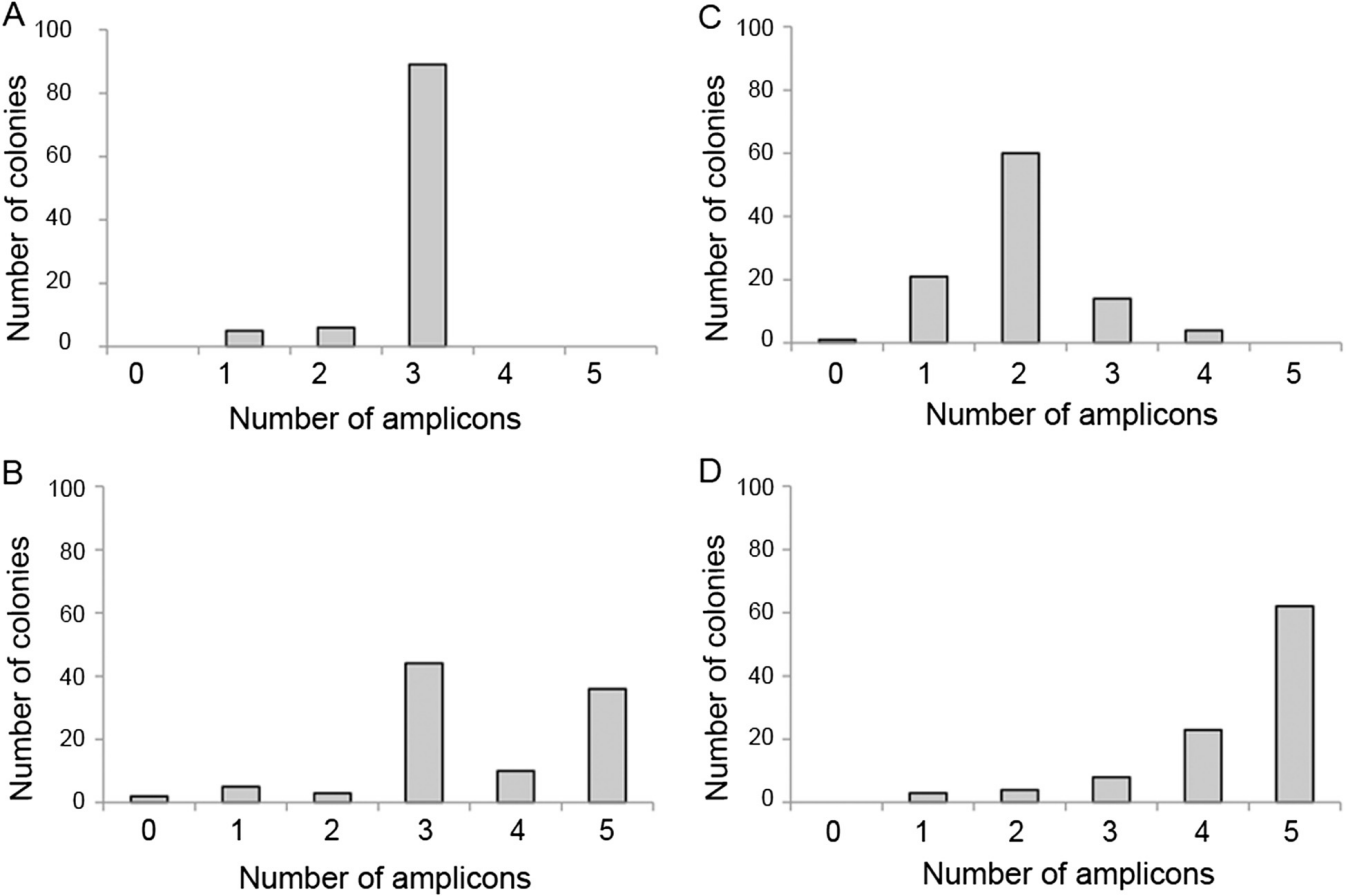
**Summary of assembly efficiency for chromosomes 25 and 26. Assembly of each of *P. tricornutum *chromosomes 25 (A and B) and 26 (C and D) from five overlapping fragments was performed using yeast spheroplast transformation.** Fragments to assemble were modified with the *URA3* selectable marker in the third fragment only (**A** and **C**) or *URA3* selectable marker in the third fragment and yeast origins of replication in fragments 2, 3, and 4 (**B** and **D**). One hundred resulting colonies were screened by low resolution multiplex PCR (one amplicon per fragment), and the number of fragments cloned for each assembly was plotted.

Resulting yeast colonies were patched several times on selective media. One hundred colonies of each assembly (i.e. with or without additional yeast replication origins) were screened by multiplex PCR in which one amplicon derived from each of the five assembled fragments. For both chromosomes 25 and 26, no complete assemblies were observed without the addition of yeast replication origins (Figure 2). In the chromosome 25 assembly with additional yeast origins of replication, 36% of the yeast colonies contained all five fragments (Figure 2). For the chromosome 26 assembly with added ARS regions, 62% of the clones contained all five fragments (Figure 2). The greatly improved efficiency of assembly using fragments that contain additional yeast origins confirms their utility for improving chromosome-scale assemblies with eukaryotic DNA (Figure 2). We also investigated the use of electroelution as a purification method for large DNA fragments. Results without gel purification of the fragments by electroelution yielded a lower percentage of correct colonies, and only 5 of 100 colonies in the chromosome 25 assembly had all five fragments.

Assembled chromosomes that were positive by PCR for all five fragments were further analyzed by high resolution multiplex PCR. In this analysis, primers were designed to amplify a band every 20 kb. The results from the high resolution multiplex PCR indicated that all regions present in the original five fragments were successfully assembled into a final molecule (Additional file 1: Figure S3). Assembled chromosome plasmids with correct multiplex PCR profiles were transformed to *E. coli* and retested with high resolution multiplex PCR to confirm that the entire plasmid was transferred.

**Figure 3:**
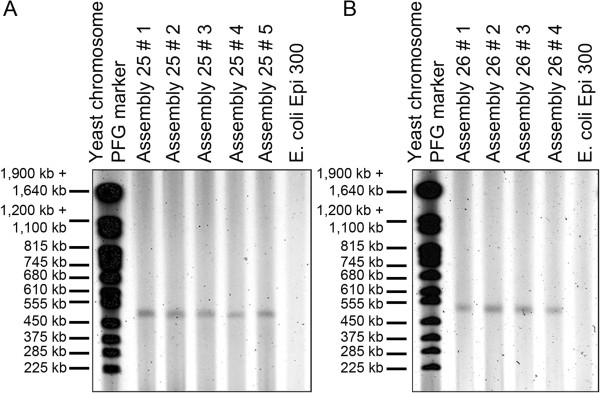
**Final assemblies of *P. tricornutum chromosomes*.** Assembled chromosomes that tested positive by high resolution multiplex PCR were transferred to *E. coli* and supercoiled plasmids were purified in agarose plugs. DNA purified in plugs from *E. coli* containing assembled chromosome 25 **(A)** or 26 **(B)** was separated by pulsed-field gel electrophoresis.

The size of the assembled chromosomes was then analyzed by pulsed field gel electrophoresis. The assembled circular *P. tricornutum* chromosomes were prepared from *E. coli* in agarose plugs to prevent shearing the large circular plasmids and were separated by pulsed field gel electrophoresis (Figure 3). DNA preparations from *E. coli* cells containing chromosome 25 yielded a single band at a size consistent with the expected size of the cloned chromosome 25 (513 kb including vector and other added sequence). Preparations from *E. coli* cells containing chromosome 26 also contained a single band at *ca*. 500 kb. This observed size was slightly larger than expected (457 kb including vector and other added sequence), but the larger observed size of the assembled molecule is consistent with the larger than expected size of fragments 26–1 and 26–2 (Figure 1).

### Manipulation of chromosome fragments in yeast

**Figure 4:**
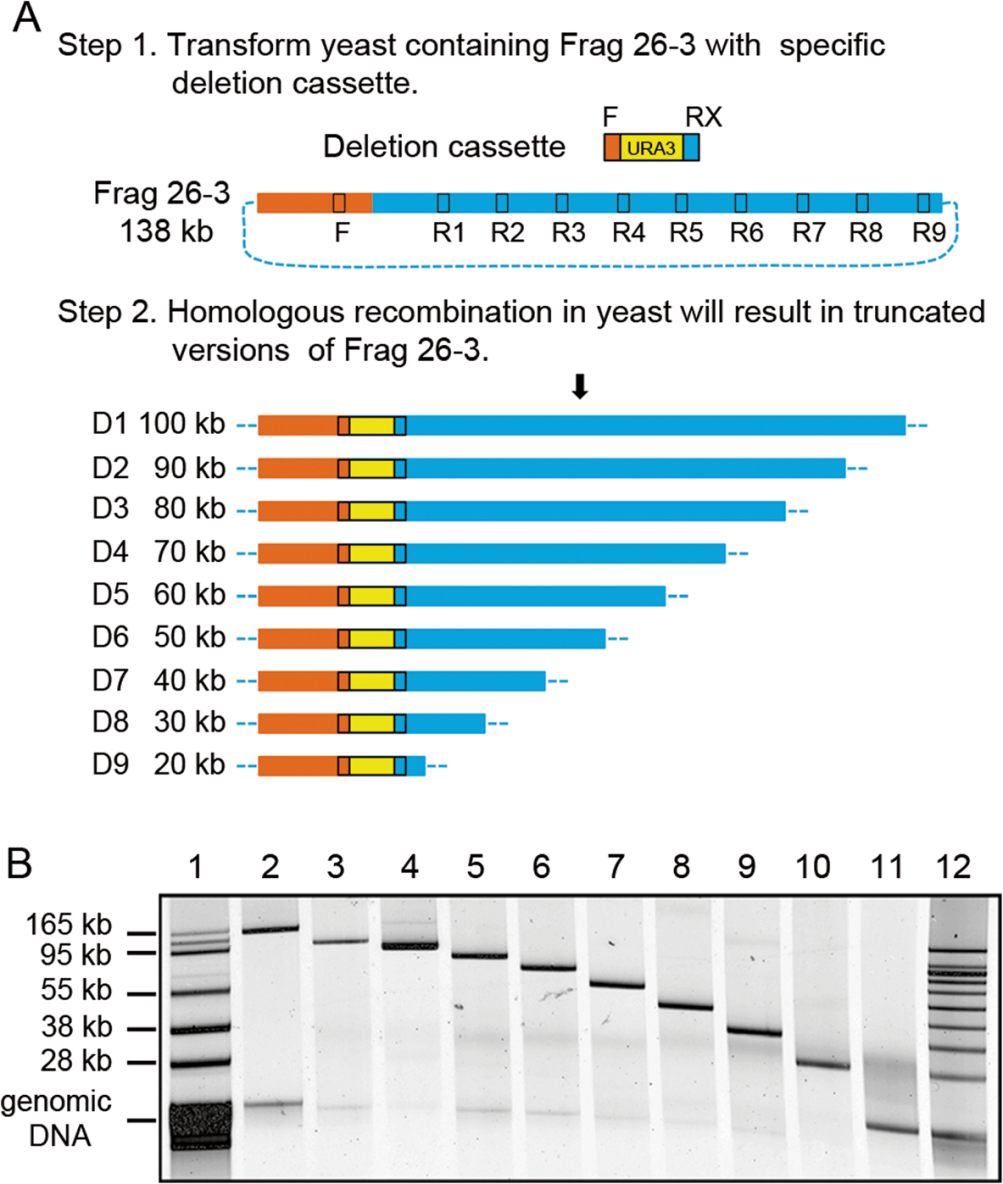
**Progressive minimization of *P. tricornutum *fragment 26–3 using yeast homologous recombination. A.** Diagram of the deletion strategy. To generate reduced versions of fragment 26–3, we inserted yeast deletion cassettes (which had 40-bp homology at the 5’ end (F) common for all deletions, 40-bp homology at the 3’ end specific for each fragment (RX where X represents homology sites 1–9), and a URA3 marker). These cassettes were PCR amplified, and yeast transformation was performed using the lithium acetate method (Step 1). Homologous recombination between the deletion cassettes and fragment 26–3 would produce desired fragments (Step 2). URA3 (yellow box), Yeast vector (orange box), *P. tricornutum* DNA representing fragment 26–3 (blue box and blue dotted line). **B.** After creation of the deletion in yeast, the plasmids were transferred to *E. coli* and purified. Supercoiled plasmids were separated by gel electrophoresis in the following order: BAC-Tracker ladder (lane 1), unmodified fragment 26–3 (lane 2), deletion of fragment 26–3 into 100 kb (lane 3), 90 kb (lane 4), 80 kb (lane 5), 70 kb (lane 6), 60 kb (lane 7), 50 kb (lane 8), 40 kb (lane 9), 30 kb (lane 10), 20 kb (lane 11).

One of the strengths of the synthetic genomic approach outlined above is the ability to manipulate target organism DNA using efficient yeast and *E. coli* genetic techniques. To demonstrate how this might be applied to the cloned *P. tricornutum* DNA, we used a mutational strategy to progressively shorten fragment 26–3. As shown in Figure 4A, a *URA3* cassette was PCR amplified to have 40-bp homology on the 5’ end to a position in the cloning vector and at the 3’ end of the *URA3* to part of the cloned 26–3 *P. tricornutum* fragment. This fragment was then transformed into yeast carrying the 26–3 plasmid to homologously recombine and replace native sequence with the *URA3* marker. Deletions from 20–100 kb were constructed using this method (Figure 4B). The deletions constructed using this method are not limited to extending from the vector as shown in Figure 4, but can be made anywhere in the fragment. Serial mutations can be easily made using techniques to recycle the *URA3* marker [28]. Once a *URA3* cassette is inserted, replacement of the URA-marked deleted region with a sequence of interest can be easily selected by plating the transformant on media containing 5-FOA [27]. After the fragments are modified as desired, they can be reassembled into entire chromosomes as described above.

## Discussion

In this work, we found that the efficiency of yeast-based assembly of entire *P. tricornutum* chromosomes was improved by adding yeast replication origins to the large fragments before assembly. Although similarly large fragments of eukaryotic DNA have been cloned as YACs in various genome projects, the increased efficiency of assembly and maintenance afforded by adding yeast replication origins may have been related to the 10% higher G + C content of *P. tricornutum* relative to yeast (48% vs. 38% G + C, respectively) (Table 1). Although sequences found in *Zea mays* (47% G + C and similar to *P. tricornutum*) function as yeast origins [16], our finding may highlight the importance of variation from the mean G + C content as well as the mean value itself in predicting whether additional yeast replication origins may improve assembly and maintenance in yeast. For example, while the human genome has an average G + C content of 41%, many small regions were found with G + C contents that vary from 33% to 55% [29], and large YACs spanning hundreds of kilobases of human DNA have been maintained in yeast. Thus, mean G + C content may not be as important as the frequency of deviation from the mean in understanding how well DNA of a particular organism will be maintained in yeast. If frequent regions of low G + C content are found, then large DNA fragments may be more efficiently cloned without addition of yeast origins.

This paper describes an improved chromosome assembly process that uses electroelution to concentrate and purify large DNA fragments to increase the efficiency of assembly. Electroelution has been used extensively to select for large inserts in BAC library construction [30-33]. Initial attempts to assemble the *P. tricornutum* DNA fragments used a “digest and mix” strategy. In this initial method, plasmids were digested to release the *P. tricornutum* fragments from the cloning vector, and the five fragments were subsequently mixed together with a new cloning vector without any purification or removal of old cloning vectors. We found that this method yielded very few colonies of which only a small percentage were correct. In this study, we describe purification of the large, digested DNA fragments by electroelution from agarose gels before assembly in yeast. This modification improved the rate of successful assembly. For electroelution, we used RECO chips (Takara). These small disposable devices consist of a chamber with permeable fabric on one side and impermeable dialysis membrane on the other side. The chip is inserted into an agarose gel after bands of interest have been separated and the DNA of interest is electrophoresed into the chip chamber. The DNA is eluted by a quick low speed centrifugation step. Although the RECO chip product is currently discontinued by the manufacturer, other electroelution techniques should suffice as they have been used successfully in the construction of large BAC libraries [31].

Assembly of chromosome 25 matched the expected size and multiplex PCR profile, and our final assembly of chromosome 26 differed from the expected size and PCR profile based on the published genome sequence. The differences between the expected size and multiplex PCR profiles were likely due to the larger-than-expected sizes of fragments 26–1 and 26–2. In the end, the assembled whole chromosome 26 was consistent with the starting component fragments indicating that the assembly process itself was robust for the *P. tricornutum* DNA. To better understand the differences between chromosomes 25 and 26, we searched for repeated regions in each chromosome using BLAST. The results showed that chromosome 25 had very little repeated DNA while chromosome 26 had a region containing several repeats. These repeats are present in a 30-kb region at the 5’ end of fragment 26–2 and contained at least 4 direct repeats each 2–4 kb in length (Additional file 1: Table S1). It is possible that during the process of cloning fragment 26–2 in yeast, different rearrangements occurred resulting in cloned regions of different sizes. However, we also cannot rule out the possibility that the different-sized fragments reflect natural diversity in this region in our *P. tricornutum* cell population. Because the *P. tricornutum* cultures are largely maintained by serial passage for long periods of time, populations of cells within the culture with different genetic characteristics may have resulted. We did not find repeat regions in fragment 26–1; however, the larger than expected size for fragment 26–1 may be explained by a gap in sequence at the 5’end of this fragment. This gap is estimated to be approximately 17 kb; however, it is possible that it is larger than 17 kb which would explain the size of fragment 26–1 which we have cloned.

Despite the differences between our assembled chromosome 26 and the expected version based on the genome sequence, our data support the fact that using the assembly techniques described in this paper we are able to accurately and efficiently assemble the pieces we had cloned. To avoid potential difficulties, future attempts to assemble eukaryotic chromosomes should perform a BLAST search to identify repeated sequences. Such a search can be easily implemented and visualized in the NCBI Genome Workbench software. If repeats are identified, attempts should be made to design a fragment cloning strategy that places the repeated sequences in the middle of the fragment and not at the junctions between two fragments as in fragment 26–2.

## Conclusion

Using *P. tricornutum* as our model eukaryote, we have demonstrated a technique for assembly, maintenance, and manipulation of entire eukaryotic chromosomes in yeast. We show that addition of yeast replication origins has improved assembly and maintenance of two eukaryotic chromosomes with moderate G + C content (47%) and that this is likely to translate to other eukaryotic DNA fragments. Because many other algae have high G + C content including the model green alga *Chlamydomonas reinhardii* (G + C content 64%)[34], application of these techniques may improve genome scale cloning in a variety of organisms. Given the challenges of slow growth and limited genetic tools in a variety of algal species of interest, developing synthetic genomic approaches may improve genetic characterization of these species.

## Methods

### Strains, media, and growth conditions

For all yeast transformations, we used *Saccharomyces cerevisiae* VL6-48 (ATCC MYA-3666: MATα his3-Δ200 trp1-Δ1 ura3-52 lys2 ade2-1 met14 cir^0^). Yeast cells were grown in rich medium (YEPD) or complete minimal (CM) medium lacking histidine and uracil or tryptophan and uracil (Teknova) at 30°C. *Escherichia coli* (Epi300, Epicentre) strains were grown on Luria broth or agar supplemented with chloramphenicol (20 mg L^-1^) or kanamycin (50 mg L^-1^) as needed. *Pheodactylum tricornutum* was grown in L1 medium [35] at 18°C under cool white fluorescent lights (50 μE m^-2^ s^-1^). Sequence information for *P. tricornutum* was taken from the DOE genome sequencing site [36] as described [20].

### Preparation of agarose-embedded DNA

DNA from *P. tricornutum* for TAR cloning of the ~100-kb fragments was isolated in agarose plugs using the Bio-Rad CHEF Genomic DNA Plug Kit with a protocol that was adapted for use with *P. tricornutum*. To prepare the plugs, 50 ml of mid-log phase (10^8^ cells ml^-1^) *P. tricornutum* culture was centrifuged at 1500 × g for 5 min at 10°C. Cells were washed once with 50 ml of 1 M sorbitol and were resuspended in 2 mL of SPEM solution (1 M sorbitol, 10 mM EDTA pH 7.5, Na_2_HPO_4_ · 7H_2_O (2.08 g L^-1^), NaH_2_PO_4_ · 1H_2_O (0.32 g L^-1^)). The cell suspension was incubated for 5 min at 37°C and mixed with an equal volume of 2.0% low-melting-point agarose in 1 × TAE buffer (40 mM Tris, 20 mM acetic acid and 1 mM EDTA) which was equilibrated at 50°C. Aliquots of 100 μl were transferred into plug molds (Bio-Rad, catalog # 170–3713) and allowed to solidify for 10 min at 4°C. Next plugs were removed from the molds into 50 ml conical tube containing 5 ml of protoplasting solution (4.56 mL of SPEM solution, 200 μl Zymolyase-100 T solution (50 mg ml ^-1^ dissolved in H_2_O), 200 μl lysozyme (25 mg ml^-1^), 40 μl β-Mercaptoethanol and incubated for 1 h at 37°C. Next, plugs were washed with 25 ml of wash buffer (20 mM Tris, 50 mM EDTA, pH 8.0), and then incubated in 5 ml in Proteinase K buffer (100 mM EDTA (pH 8.0), 0.2% sodium deoxycholate, and 1% sodium lauryl sarcosine, 1 mg ml^-1^ Proteinase K) for 24 hr at 50°C. After proteinase K treatment, plugs were washed according to the Bio-Rad CHEF-Dr III manual.

### Construction of cloning vectors used in this study

Vectors used for cloning and assembly of the ~100 kb fragments and entire *P. tricornutum* chromosomes were constructed from the template plasmids pBK-RBYV-HIS3URA3 and pBK-RBYV-TRP1URA3 using yeast assembly methods [17,37]. Maps and sequences for both vectors are available in the Supplementary Information. Both vectors contained the *URA3* gene from pYAC-RC (ATCC 37610). For the vector pBK-RBYV-HIS3URA3, the BACYAC backbone pCC1BACHIS3 was used containing the *HIS3* gene [17]. For pBK-RBYV-TRP1URA3, the HIS3 gene was replaced with the *TRP1* gene from pRS314 (GenBank Accession U03440). Both pBK-RBYV-HIS3URA3 and pBK-RBYV-TRP1URA3 contain the *ShBle* cassette from pAF6 [38] and the conjugative transfer origin (OriT) from pRL2948a (C. P. Wolk, unpublished data). Both vectors were designed to have I-CeuI and XhoI restriction sites at the junction between OriT and *URA3*, and at the junction of the *URA3* and pCC1BAC backbone, XhoI and I-SceI sites were inserted.

Specific vectors for cloning each of the ~100 kb *P. tricornutum* DNA fragments by TAR cloning were assembled in yeast by co-transforming XhoI-digested template plasmids pBK-RBYV-HIS3URA3 and two 200-bp PCR-amplified *P. tricornutum* DNA homology regions. The vector pKB-RBYV-HIS3URA3 was designed so that digestion with XhoI would excise the *URA3* cassette. Our strategy to create 200-bp homology regions on either side of the *URA3* cassette was to reassemble the two pieces of the digested template vector with the 200-bp homology regions inserted on either side of the *URA3* cassette. Each of the 200-bp *P. tricornutum* PCR fragments contained 25–40 bp homologies to the template vector (pBK-RBYV-HIS3URA3) on either side of an XhoI site. The resulting plasmid contained the *URA3* cassette cloned between two 200-bp *P. tricornutum* homology regions that specified the boundaries of the resulting TAR-cloned fragment, and the pBK-RBYV-HIS3 backbone. Primers used to amplify each of the 200-bp *P. tricornutum* homology regions are listed in Additional file 1: Table S4.

The design of the primers used to amplify the 200-bp homology regions was such that after assembly only one of the XhoI sites would be restored and the second XhoI site would be replaced with a PacI site. Digestion with either XhoI or PacI would allow the vector to be linearized while maintaining the *URA3* marker which could be used as negative selection to select for double recombination events in which both homology regions had recombined with their target regions in the *P. tricornutum* chromosome. Alternatively each vector could be digested with both XhoI and PacI to remove *URA3* marker before TAR-cloning. We used both approaches in this paper and found that the highest cloning efficiency of target genomic fragments that were cut only on one side (e.g. fragment 25–1) was obtained using vector that had been cut at both ends to release *URA3*, and therefore the negative selection could not be applied. For increased yield, vectors verified by PCR as correctly assembled were moved from yeast to *E.coli* and were purified by alkaline lysis before being digested with XhoI and/or PacI [39].

Vectors for assembling entire chromosomes 25 and 26 were constructed in yeast by co-transforming XhoI-digested template pBK-RBYV-TRP1URA3 and two 500-bp PCR amplified homology regions. The first homology region matched the 5’ region of the cloned *P. tricornutum* sequence in fragments 25–1 or 26–1. The second homology region matched the 3’ region of the cloned *P. tricornutum* sequence in pieces 25–5 or 26–5. The first homology region had 35 bp overlap with the template vector at the XhoI site near the OriT region, and the second 500 bp region had 35 bp overlap at the XhoI site near the pCC1BAC sequence. In addition, the 3’ end of homology region one contained 35 bp overlap with 5’ end of homology region two so that the two pieces could be assembled together in a vector. The design of the primers used to amplify the homologous regions was such that after assembly an XhoI site would be created at the junction of homology region one and two. This allowed for digestion of the resulting vector with XhoI to linearize and expose the two homology regions before assembly of the entire chromosome. For chromosome 25, the first 500-bp homology region was amplified by primers Pt25-AV15-1 and Pt25-AV15-2new and the second homology region was amplified using primers Pt25-AV15-3new and PT25-AV15-4. For chromosome 26, the first 500-bp homology region was amplified by primers Pt26-av-15-F1 and Pt26-av-15-R1 and the second region was amplified by primers Pt26-av-15-F2 and Pt-av-15-R2 (See supplementary information).

### TAR-cloning 100-kb fragments and assembly of full length chromosomes

*P. tricornutum* DNA for TAR cloning was prepared in agarose plugs as described above and in-plug digestion was performed as previously described [17] using specific enzymes listed in Table 2. After digestion, plugs were washed in TE buffer (pH 8) and were melted in 100 μl TE buffer at 65°C for 10 min. The molten agarose was then equilibrated at 42°C and 2 μl of β-agarase (NEB) was added and incubated for 8 h. 10 μl of DNA from plugs, (typically 100 ng/μl ) was mixed with 300–500 ng of vector and the assembly was performed by mixing the DNA with yeast spheroplasts prepared as previously described [17,37]. Resulting colonies were screened by PCR using primers specific to a region in the cloned piece (see multiplex primers below). Fragments 25–1, 25–5, 26–1, and 26–5 were further screened by PCR to verify that the proper sub-telomeric region was cloned using the following primer pairs, respectively: Endchk-F + Pt25-1endchk-R, Endchk-R + Pt25-5endchk-F2, Endchk-F + Pt26-1endchk-R, Endchk-R + Pt26-5endchk-F2 (See Additional file 1: Table S4). Plasmids from positive clones were isolated by alkaline lysis and transformed into *E. coli* for further analysis and confirmation of size by separation with agarose gel electrophoresis.

To assemble entire *P. tricornutum* chromosomes, plasmids containing the cloned fragments were purified from *E. coli* using an alkaline lysis procedure [39]. Each of the five fragments (20–30 μg plasmid) composing either chromosome 25 or 26 was excised from the cloning vector by digest with I-SceI and I-CeuI for 8 h at 37°C in a total volume of 50 μl. These excised fragments were either precipitated and used directly for assembly or subjected to gel purification and electroelution. For gel purification, the entire 50 μl digest was loaded on a 1% agarose gel in 1X Tris-Acetate-EDTA (TAE) buffer and was separated by electrophoresis for 8 h at 80 V at 4°C. The gel was then stained briefly with ethidium bromide, and the excised fragments were each separated from genomic DNA and vector by electroelution using RECO chips (Takara). To capture the large DNA fragments in the chips, the gel was rotated 90° and a RECO chip was inserted into the gel so that the large *P. tricornutum* DNA fragments would electrophorese into the chip for 1 h at 100 V at room temperature. DNA was eluted from the chip by a centrifugation for 10 sec (1,500 × g), and final concentrations of DNA were typically between 20–30 μg μl^-1^.

Digested fragments were mixed with 500–1000 ng of vector and transformed into yeast spheroplasts as previously described [37]. Typically, 3–5 μg of each fragment was used for assemblies of fragments without electroelution and 100–500 ng of each fragment was used for electroeluted samples. Resulting yeast colonies were screened by colony PCR using a multiplex primer set containing one amplicon on each assembled fragment using the Multiplex PCR Kit (Qiagen). Primers used for multiplex PCR can be found in Additional file 1: Table S4 and are listed as “low resolution multiplex PCR”. Plasmids from clones positive for all five amplicons were isolated by alkaline lysis and transformed into *E. coli* strain Epi300. Further analysis of the clones was performed on plasmids isolated from *E. coli* including high resolution multiplex PCR and CHEF gel analysis on the whole assembled chromosomes. High resolution multiplex PCR consisted of amplicons designed every ~20 kb along the chromosome. Primers for this can be found in Additional file 1: Table S4 and were pooled according to Additional file 1: Table S3. The reactions were performed using the Qiagen Multiplex PCR kit according to the manufacturer’s instructions. Amplicons were separated by agarose gel electrophoresis using 2% agarose gels made with 1X TAE. CHEF gel analysis of assembled *P. tricornutum* chromosomes was performed as previously described for other cloned large plasmids [17]. Briefly, assembled *P. tricornutum* chromosomes propagated in *E. coli* were isolated in agarose plugs using the CHEF Genomic DNA Plug Kit according to the manufacturer’s instructions.

### Manipulation of fragments

Yeast replication origins were inserted at defined locations in fragments 2, 3, and 4 of chromosomes 25 and 26 (Additional file 1: Table S2). The *ARSH3* sequence from yeast was fused to a sequence encoding the kanamycin antibiotic resistance marker from pACYC177 and recombined into the cloned *P. tricornutum* fragments using lambda red recombineering as previously described [17]. The KanR-ARS fragment was amplified by PCR using primers described in Additional file 1: Table S2. Positive colonies were screened by PCR using primers flanking the insertion site and the increase in amplicon size based on insertion of the KanR-ARS fragment indicated a correctly-recombined clone (Additional file 1: Table S2). Positive clones were purified, confirmed to have the correct size by agarose gel electrophoresis and used in assembly reactions.

The strategy to modify the fragments in yeast was based on insertion of the *URA3* gene using homologous recombination. This strategy was used to insert the *URA3* gene in the middle of fragments 25–3 and 26–3 to assist in proper assembly, as well as to shorten fragment 26–3 to make a series of deletions. The *URA3* gene with appropriate homology regions was amplified from pYAC-RC vector (ATCC 37610). For the insertions into fragment 25–3 using primers URApt25-3_F + URApt25-3_R, and into fragment 26–3 using primers URApt26-3_F + URApt26-3_R (See Additional file 1: Table S2). For deletions in fragment 26–3, the *URA3* gene was amplified using a common forward primer for each insertion and a unique reverse primer that specified the insertion site and the degree of deletion created (e.g. URA-F + URA-R100, etc., see Additional file 1: Table S4). The amplified *URA3* cassettes were transformed into yeast carrying Fragment 25–3 or 26–3 using lithium acetate transformation [40]. Yeast colonies were selected on media lacking histidine and uracil, and correct clones were identified by PCR at either side of the *URA3* insertion junction. The junction at the 5’ end of the insertion (i.e. the “left” side) was common to all plasmids generated in the deletion series and was screened using the primers F-left + R-left). The junction at the 3’ end of the URA3 insertion (i.e. the “right” side) was screened using a common forward primer (F-right) and a primer that denoted the final size of the resulting plasmid (100-R-right, etc., See Additional file 1: Table S4). Fragments identified as having the correct insertion by PCR were moved from yeast to *E.coli* and plasmids were purified and further tested by agarose gel electrophoresis to verify correct plasmid size.

## Abbreviations

BAC-YAC: Bacterial artificial chromosome-yeast artificial chromosome; TAR: Transformation-associated recombination.

## Competing interests

JCV. is Chief Executive Officer and Co-Chief Scientific Officer of Synthetic Genomics, Inc. HOS. is Co-Chief Scientific Officer and on the Board of Directors of Synthetic Genomics, Inc. CAH. is Chairman of the Synthetic Genomics, Inc. Scientific Advisory Board. All three of these authors and the J. Craig Venter Institute hold Synthetic Genomics, Inc. stock.

## Authors’ contributions

The following authors designed experiments and analyzed data: BJK, BM, JJ, WJH, YCL, CT, ITY, VNN, RYC, AEA, CLD, JIG, CAH, HOS, JCV, and PDW. Experiments were performed by BJK, BM, JJ, WJH, YCL, CT, ITY, and PDW. The paper was written by BJK and PDW. All authors read and approved the final manuscript.

## Supplementary Material

Additional file 1 Supplemental Figures, Tables, and Plasmid sequences used in this study. Click here for file
