# Supplementary material for: Assembly of eukaryotic algal chromosomes in yeast
Source: J Biol Eng. 2013 Dec 10;7:30. doi: 10.1186/1754-1611-7-30 (PMC4029449; doi:10.1186/1754-1611-7-30)
Supplement: Additional file 1 — Supplemental Figures, Tables, and Plasmid sequences used in this study. [file 1754-1611-7-30-S1.docx]

# Supplementary Information

**
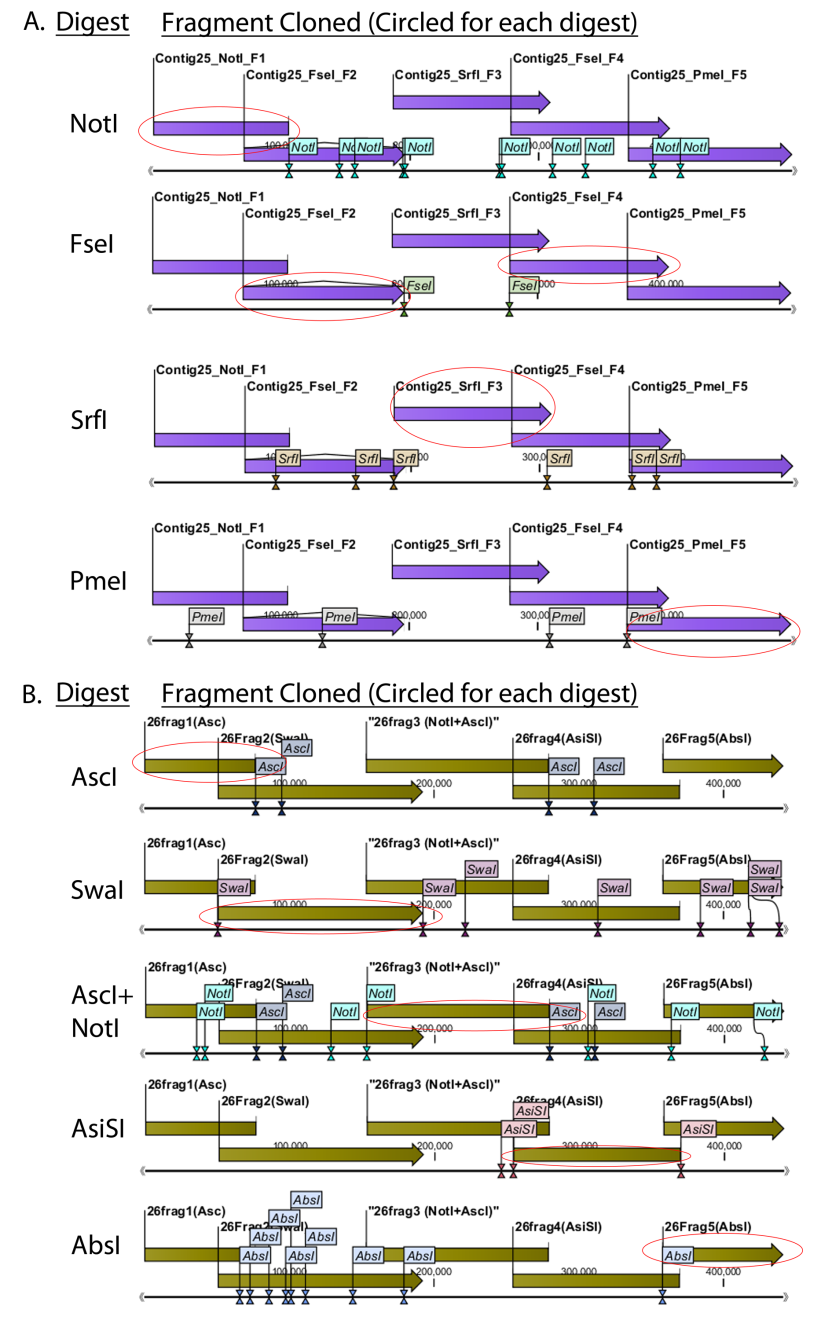
**

**Figure S1**:

Map of restriction enzyme digests used in cloning the *ca*. 100 kb *P. tricornutum* fragments. Agarose plugs containing *P. tricornutum* DNA were digested with the enzymes listed in the figure and the fragments targeted with each digest are circled in red. Note that some enzymes only cut at one end of a given fragment as discussed in the text. **A.** Chromosome 25 fragments. **B.** Chromosome 26 fragments.





**Figure S2**:

High resolution multiplex PCR results for cloned ~100 kb *P. tricornutum* fragments. Amplicons were designed every ~20 kb for chromosome 25 (A) and chromosome 26 (B). Amplicons increase in size with increasing distance from the 5’ end of the chromosome for a given multiplex primer set and the multiplex primer sets continue in order along the chromosome length. Fragments cloned from chromosome 25 were tested using 3 different multiplex mixes and fragments from chromosome 26 were tested using 2 different multiplex mixes. Bands missing from the cloned fragments but found in the *P. tricornutum* gDNA control are indicated by arrows. Faint band visible in A, MPX2, lane 2, below the 615bp marker is most likely non-specific amplification which we have previously observed in cases where multiplex primers are used (in this case 18 different primers in one reaction mixture) and where there are few target sites (only one in this case).





**Figure S3**:

High resolution multiplex PCR results for assembled *P. tricornutum* chromosomes 25 (A) and 26 (B). Five independently assembled clones from each of chromosomes 25 and 26 were tested using the primers described in Supplementary Fig. 2.

**Table S1: Repeated sequence in fragment 26-2 found in the published genome sequence.**

| **Repeat number** | **Start position (in bp from beg of chrom 26)** | **End position (in bp from beg of chrom 26)** | **Length (in bp)** | **% Identity** |
| --- | --- | --- | --- | --- |
| 1 | 70802 | 72904 | 2103 | 99% |
| 2 | 76515 | 78973 | 2459 | 99% |
| 3 | 95006 | 99367 | 4632 | 99% |
| 4 | 98696 | 103275 | 4640 | 99% |

**Table S2: Primers used in the amplification and screening of 200bp-homology regions, KanR-ARS, and *URA3* insertions. Primer sequences can be found in Supplementary Table S4.**

| **Frag** | **Primers to amplify 200-bp homology region 1** | **Primers to amplify 200-bp homology region 2** | **Primers to amplify KanR-ARS** | **Primers to screen KanR-ARS insertion** | **Primers to amplify *URA3*** | **Primers to screen for *URA3* insertion** |
| --- | --- | --- | --- | --- | --- | --- |
| 25-1 | 1.4-25.1_1F + 1.4-25.1_1R | 1.4-25.1_2F + 1.4-25.1_2R |  |  |  |  |
| 25-2 | 1.4-25.2_1F + 1.4-25.2_1R | 1.4-25.2_2F + 1.4-25.2_2R | 25Red2-F + 25Red2-R | 25-Screen-2F + 25-Screen2R |  |  |
| 25-3 | 1.4-25.3_1F + 1.4-25.3_1R | 1.4-25.3_2F + 1.4-25.3_2r | 25Red3-F + 25Red3-R | 25-Screen-3F + 25-Screen-3R | URApt25-3_F + URApt25-3_R | 25-urascreenF + 26-3urascreenR2 |
| 25-4 | 1.4-25.4_1F + 1.4-25.4_1R | 1.4-25.4_2F + 1.4-25.4_2R | 25Red4-F + 25Red4-R2 | 25-Screen-4F + 25-Screen-4R |  |  |
| 25-5 | 1.4-25.5_1F + 1.4-25.5_1R | 1.4-25.5_2F + 1.4-25.5_2R |  |  |  |  |
| 26-1 | Pt26-1-1F + Pt26-1-1R | Pt26-1-2F + Pt26-1-2R |  |  |  |  |
| 26-2 | Pt26-2-1F + Pt26-2-1R | Pt26-2-2F + Pt26-2-2R | 26Red2-F2 + 26Red2-R2 | 26-2-screenF + 26-2-screenR |  |  |
| 26-3 | Pt26-3-1F + Pt26-3-1R | Pt26-3-2F + Pt26-3-2R | 26Red3-F3 + 26Red3-R3 | 26red3checkF3 + 26red3checkR3 | URApt26-3_F + URApt26-3_R | 26-urascreenF3+25-urascreenR2 |
| 26-4 | Pt26-4-1F + Pt26-4-1R | Pt26-4-2F + Pt26-4-2R | 26Red4-F2 + 26Red4-R2 | 26-4-screenF + 26-4-screenR |  |  |
| 26-5 | Pt26-5-1F + Pt26-5-1R | Pt26-5-2F + Pt26-5-2R |  |  |  |  |

**Table S3: Primers used in High Resolution Multiplex PCR reactions**

| **25 MPX 1** | **25 MPX 2** | **25 MPX 3** | **26 MPX 1** | **26 MPX 2** |
| --- | --- | --- | --- | --- |
| pt25set1F1  pt25set1R1  pt25set1F2  pt25set1R2  pt25set1F3  pt25set1R3  pt25set1F4  pt25set1R4  pt25set1F5  pt25set1R5  pt25set1F6  pt25set1R6  pt25set1F7  pt25set1R7  pt25set1F8  pt25set1R8  pt25set1F9  pt25set1R9 | pt25set2F1  pt25set2R1  pt25set2F2  pt25set2R2  pt25set2F3  pt25set2R3  pt25set2F4  pt25set2R4  pt25set2F5  pt25set2R5  pt25set2F6  pt25set2R6  pt25set2F7  pt25set2R7  pt25set2F8  pt25set2R8  pt25set2F9  pt25set2R9 | pt25set3F1  pt25set3R1  pt25set3F2  pt25set3R2  pt25set3F3  pt25set3R3  pt25set3F4  pt25set3R4  pt25set3F5  pt25set3R5  pt25set3F6  pt25set3R6  pt25set3F7  pt25set3R7  pt25set3F8  pt25set3R8 | Pt26set1_F1  Pt26set1_R1  Pt26set1_F2  Pt26set1_R2  Pt26set1_F3  Pt26set1_R3  Pt26set1_F4  Pt26set1_R4  Pt26set1_F5  Pt26set1_R5  Pt26set1_F6  Pt26set1_R6  Pt26set1_F7  Pt26set1_R7  Pt26set1_F8  Pt26set1_R8  Pt26set1_F9  Pt26set1_R9  Pt26set1_F10  Pt26set1_R10  Pt26set1_F11  Pt26set1_R11  Pt26set1_F12  Pt26set1_R12 | Pt26set2_F1  Pt26set2_R1  Pt26set2_F2  Pt26set2_F2  Pt26set2_F3  Pt26set2_R3  Pt26set2_F4  Pt26set2_R4  Pt26set2_F5  Pt26set2_R5  Pt26set2_F6  Pt26set2_R6  Pt26set2_F7  Pt26set2_R7  Pt26set2_F8  Pt26set2_R8  Pt26set2_F9  Pt26set2_R9 |

**Table S4: Primers used in this study**

| 1.4-25.1_1F | CAAGACGATCCGTAACTATAACGGTCCTAAGGTAGCGAACTTTTGGATGTGTTCCAAGCG |
| --- | --- |
| 1.4-25.1_1R | ATCTACCTGCCTGGACAGCATGGCCTGCAACGTTAATTAATGACATAACTCCCTGTAATT |
| 1.4-25.1_2F | acccgtatcgtgagcatcctctctcgtttcatcgCTCGAGTTCCTGGACGAGTCCGTCTT |
| 1.4-25.1_2R | GGGCAACCAGCTATATTACCCTGTTATCCCTAGCGTAACTGCCGCTTCTTCCGAGTGGCG |
| 1.4-25.2_1F | CAAGACGATCCGTAACTATAACGGTCCTAAGGTAGCGAACAGATCTCGTCATAGTTAACG |
| 1.4-25.2_1R | ATCTACCTGCCTGGACAGCATGGCCTGCAACGTTAATTAAAGAGATACCAAAGGATTTCC |
| 1.4-25.2_2F | cccgtatcgtgagcatcctctctcgtttcatcgCTCGAGCAAAAGCAGGCGCATTCGCG |
| 1.4-25.2_2R | GGGCAACCAGCTATATTACCCTGTTATCCCTAGCGTAACTCCGGCCGCACTGTCTCGTTG |
| 1.4-25.3_1F | CAAGACGATCCGTAACTATAACGGTCCTAAGGTAGCGAAGGGCAGCGCGGGACGTCGCTT |
| 1.4-25.3_1R | ATCTACCTGCCTGGACAGCATGGCCTGCAACGTTAATTAACAGACCCTTTACAAGCTCCG |
| 1.4-25.3_2F | acccgtatcgtgagcatcctctctcgtttcatcgCTCGAGAACACTCTGAAGCCCCAACC |
| 1.4-25.3_2r | GGGCAACCAGCTATATTACCCTGTTATCCCTAGCGTAACTGGGCTAGACATGATGGCATG |
| 1.4-25.4_1F | CAAGACGATCCGTAACTATAACGGTCCTAAGGTAGCGAACCCTATTGCTTACGTTCCTAG |
| 1.4-25.4_1R | ATCTACCTGCCTGGACAGCATGGCCTGCAACGTTAATTAAGTGTGCAGTAATCGGCAGGC |
| 1.4-25.4_2F | acccgtatcgtgagcatcctctctcgtttcatcgCTCGAGGTCAACTCCTCCAACGTCAC |
| 1.4-25.4_2R | GGGCAACCAGCTATATTACCCTGTTATCCCTAGCGTAACTCAGGCCAACAAGGTCAAGCA |
| 1.4-25.5_1F | CAAGACGATCCGTAACTATAACGGTCCTAAGGTAGCGAACAAACAAATATGTGGTAAAAC |
| 1.4-25.5_1R | ATCTACCTGCCTGGACAGCATGGCCTGCAACGTTAATTAAGCATCTTGAATCTCAAATTT |
| 1.4-25.5_2F | acccgtatcgtgagcatcctctctcgtttcatcgCTCGAGCCATCCCCCAAGCAAACGCG |
| 1.4-25.5_2R | GGGCAACCAGCTATATTACCCTGTTATCCCTAGCGTAACTTAGAGTTAAATATATGGGCC |
| Pt26-1-1F | CAAGACGATCCGTAACTATAACGGTCCTAAGGTAGCGAATGTGTTTCCTCAGGAGGTTAA |
| Pt26-1-1R | TCATCTACCTGCCTGGACAGCATGGCCTGCAACGCTCGAGCCAAGTTACCATCGCGATCC |
| Pt26-1-2F | CCGTATCGTGAGCATCCTCTCTCGTTTCATCG ttaattaaGCTCCGACGAGTTTGGAAAC |
| Pt26-1-2R | GGGCAACCAGCTATATTACCCTGTTATCCCTAGCGTAACTCGCGCCAAAAAAATCACTCC |
| Pt26-2-1F | CAAGACGATCCGTAACTATAACGGTCCTAAGGTAGCGAAAAATTATTCCTGTCTTTGGA |
| Pt26-2-1R | TCATCTACCTGCCTGGACAGCATGGCCTGCAACGCTCGAGCCATCAGCCCTCTCGTGGCT |
| Pt26-2-2F | CCGTATCGTGAGCATCCTCTCTCGTTTCATCG ttaattaaCGGAGTATGCCGAACTTATG |
| Pt26-2-2R | GGGCAACCAGCTATATTACCCTGTTATCCCTAGCGTAACTAAATAAGCAGCGCTCAATTC |
| Pt26-3-1F | CAAGACGATCCGTAACTATAACGGTCCTAAGGTAGCGAAGGCCGCCCCAAAAGACGTGG |
| Pt26-3-1R | TCTACCTGCCTGGACAGCATGGCCTGCAACGCTCGAGCTACCTACTGAAAAAACAAAACG |
| Pt26-3-2F | CCGTATCGTGAGCATCCTCTCTCGTTTCATCG ttaattaaCATCCAACTCTCCCCGTTGA |
| Pt26-3-2R | GGGCAACCAGCTATATTACCCTGTTATCCCTAGCGTAACTCGCGCCAGAAAAGGCGGAAG |
| Pt26-4-1F | CAAGACGATCCGTAACTATAACGGTCCTAAGGTAGCGAAATCGCTTGGCTCTGCTTATT |
| Pt26-4-1R | TCATCTACCTGCCTGGACAGCATGGCCTGCAACGCTCGAGTGCACTCGTTTCTTTATTGC |
| Pt26-4-2F | CCGTATCGTGAGCATCCTCTCTCGTTTCATCG ttaattaaAAATCCGCATCCGTCCCGTT |
| Pt26-4-2R | GGGCAACCAGCTATATTACCCTGTTATCCCTAGCGTAACTATCGCGCGGTCCCAAACTTC |
| Pt26-5-1F | CAAGACGATCCGTAACTATAACGGTCCTAAGGTAGCGAATCGAGGCCATGAATGAGATT |
| Pt26-5-1R | ATCTACCTGCCTGGACAGCATGGCCTGCAACGCTCGAGATTTACAGATCAAAGCCATCGC |
| Pt26-5-2F | CCGTATCGTGAGCATCCTCTCTCGTTTCATCG ttaattaaTGGTTTTACTTGTCCCCCAC |
| Pt26-5-2R | GGGCAACCAGCTATATTACCCTGTTATCCCTAGCGTAACTTAGCAAGGGGCTTGCCAGTG |
|  | **Low Resolution Multiplex test for 25 fragments:** |
| Nmpx25_F1 | ACACTGGAGGACGACAATCC |
| Nmpx25_R1 | CGCCTCTACTCTGGAAATGC |
| Nmpx25_F2 | ATGCCCTCCGAAATCTTTTT |
| Nmpx25_R2 | CCAAAGCAGACAAACGTTGA |
| Nmpx25_F3 | TTGGGATGCTGAACAATCAA |
| Nmpx25_R3 | TGGTCAGATGCACTTTGCTC |
| Nmpx25_F4 | ATTTCCCATACATGCCGAGA |
| Nmpx25_R4 | AAACGATCACCGTCAACACA |
| Nmpx25_F5 | ATCACATGGCCTTTGTAGCC |
| Nmpx25_R5 | TATTGAAGCCGATGGTAGCC |
|  | **Low Resolution Multiplex test for chromosome 26 fragments:** |
| Pt26M-1F | GCCGTTGAGATTGAGCATGCG |
| Pt26M-1R | ACAACGTACAAGGGACAATTCCCGT |
| Pt26M-2F | CCCTACAGGTCTTCGATAGATTCG |
| Pt26M-2R | CGTATCCCTGGAGTTTCCGACCAC |
| Pt26M-3F | GAATACACACCGTGCCCGTAGTAC |
| Pt26M-3R | GGAAGGTCCGGTAATCGTTCGTA |
| Pt26M-4F | TAGCTGACTCGGGTTCATCCTC |
| Pt26M-4R | GCGTTGACCAGATCAGAAGCTG |
| Pt26M-5F | GATGCCACAAGAGTGTCAGCTCC |
| Pt26M-5R | GGGTGTTACTGTCCGTGACTTG |
|  | **High Resolution Multiplex test for chromosome 25 fragments:** |
| pt25set1F1 | CAGCAGCAGCTTCGTTACAG |
| pt25set1R1 | TCACAAAGATTTGCCCACAA |
| pt25set1F2 | TGGACTGAACAACGGATTGA |
| pt25set1R2 | ACAACACTCCCCACAGGGTA |
| pt25set1F3 | ATGTTTTGGCTGGACTCGAC |
| pt25set1R3 | GAAACGGAGCCATGTTTGTT |
| pt25set1F4 | TCTGCTCAGATGATGGCAAG |
| pt25set1R4 | GGAACCCCAATCGGAGTAAT |
| pt25set1F5 | AACTTGCAGCTGTCGATGTG |
| pt25set1R5 | CAAAACGTGGTGATGGACAG |
| pt25set1F6 | AGAGAGCGAAGTACCCGACA |
| pt25set1R6 | CCGTGCACAACGGTATGTAG |
| pt25set1F7 | CAAATTTTACCGCCCTTCAA |
| pt25set1R7 | GCGTGAACACAGCGGTAGTA |
| pt25set1F8 | CAATCCGGTAACGAGGAAGA |
| pt25set1R8 | CACATTCCGTCACATTCCTG |
| pt25set1F9 | GAAATCCGTATCGTCGTCGT |
| pt25set1R9 | ACTCTGGCTTTCCGTTCTGA |
| pt25set2F1 | AGTGTTTGCATTTCGTGCTG |
| pt25set2R1 | GATGTTAACGCAGCAGTGGA |
| pt25set2F2 | CTGCAACTGCAACAGTCGAT |
| pt25set2R2 | CTACGGTGCCGAGGCTAATA |
| pt25set2F3 | TGAATCATGGCTGAGACCAA |
| pt25set2R3 | GTTTTGCGTTGGAAAAGGAA |
| pt25set2F4 | TAGTTCAGTACCCCGGCAAG |
| pt25set2R4 | GAATGAGCGGAGCTAACTGG |
| pt25set2F5 | CCTCCATTGCTCGTTTGATT |
| pt25set2R5 | CACGCATTTACTGCAGCCTA |
| pt25set2F6 | TACCTATCAACCCGCGTAGG |
| pt25set2R6 | TTCGCCGATCATTACAACAA |
| pt25set2F7 | TCCTTTAGGGAACGGAGGTT |
| pt25set2R7 | TTTGAGCAAGGCTTTTTCGT |
| pt25set2F8 | GCGCAATGTAGCTACGTGAA |
| pt25set2R8 | TTCAAAAATCGGACCCTCTG |
| pt25set2F9 | TCAATAATGGGACGCACGTA |
| pt25set2R9 | CTTCCCGGAGAACCACTGTA |
| pt25set3F1 | ACGAACCTCGTGTGGAAAAC |
| pt25set3R1 | CCTTGGCCTGAGTAGAGCAG |
| pt25set3F2 | CCAGGAATCGCACTAGCTTC |
| pt25set3R2 | TTCGTTCGCTATGTGCTTTG |
| pt25set3F3 | GGACCCGAGTATGCGATTTA |
| pt25set3R3 | CGAATCATACGGTTGCATTG |
| pt25set3F4 | CACCGTAAGGAGGAATCGAA |
| pt25set3R4 | CATCATCGTTGCACAGTTCC |
| pt25set3F5 | CAACGAACAGCAGGTGAGAA |
| pt25set3R5 | ACGAATCCGCAATCTAATGG |
| pt25set3F6 | GTTACCGACGGAGTTTGGAA |
| pt25set3R6 | CTAGGTGGCGTTTGGTGTTT |
| pt25set3F7 | CCTGTTGTGATGCGAAAAGA |
| pt25set3R7 | TGCTTCTGACTCGGTGTGTC |
| pt25set3F8 | CTCCGTGTTGAGGAATGGTT |
| pt25set3R8 | TTCATATTGGTGGGGACGAT |
|  | **High Resolution Multiplex test for chromosome 26 fragments:** |
| Pt26set1_F1 | GATCGCGATGGTAACTTGGT |
| Pt26set1_R1 | TTCCTGCACATTACCGTCTG |
| Pt26set1_F2 | GAGCTGGAATATTGGGGTCA |
| Pt26set1_R2 | GAGTCGTCGAAGCGGAATAG |
| Pt26set1_F3 | ACATTTTGCCAGACGTTTCC |
| Pt26set1_R3 | TTCTGCTGTTCCGTGAGTTG |
| Pt26set1_F4 | TCTGCCCATACAGGACACAA |
| Pt26set1_R4 | GCTCGGTCGCCTAATGTAAA |
| Pt26set1_F5 | ACTTGTCGCTCCATCAGCTT |
| Pt26set1_R5 | ATGGCGTTGTGGTAAAGGTC |
| Pt26set1_F6 | GGACTGTCCGATGTGGAGTT |
| Pt26set1_R6 | CGTAGTATTTCGCCCACGTT |
| Pt26set1_F7 | CTCATCAATTTCCGGTGCTT |
| Pt26set1_R7 | AGTCGAAGTGCGGAAAAGAA |
| Pt26set1_F8 | AACGTCAAACACCATGACGA |
| Pt26set1_R8 | CAAAACTTCCCCCGACTACA |
| Pt26set1_F9 | ATTGGAGCAGGCTTGACAGT |
| Pt26set1_R9 | GGTGCCACAACCCAAGTAGT |
| Pt26set1_F10 | AGTACCGTATCGACGGGTTG |
| Pt26set1_R10 | TCGGGTAACGCGTATCCTAC |
| Pt26set1_F11 | CAAACAGCGTGAAAACCTCA |
| Pt26set1_R11 | ACTTCGAACGCCTATCATGG |
| Pt26set1_F12 | GTCGGTTGGCATTCTTGATT |
| Pt26set1_R12 | CAGCAGAAATTGAAGGCACA |
| Pt26set2_F1 | CGCAAACTCTTGACGATTCA |
| Pt26set2_R1 | TTCCTTTAAACCGTCCATGC |
| Pt26set2_F2 | CAGCTGACGTTGGATTGAGA |
| Pt26set2_F2 | TGGAATCGACCGGATCTTAC |
| Pt26set2_F3 | CATTTGCGCCCATAGTTTTT |
| Pt26set2_R3 | ACTTGGCATCCGACCTACTG |
| Pt26set2_F4 | GCGTTCTGCGAGAGTATTCC |
| Pt26set2_R4 | GGGTGTTCCCAGACTTTCAA |
| Pt26set2_F5 | ACTGCGTGGAACCTTATTGG |
| Pt26set2_R5 | GGACACGTGCAGACAGGTAA |
| Pt26set2_F6 | AATGAAGGAACCGACGTCAC |
| Pt26set2_R6 | TGCGTTCAAAGACGAACAAG |
| Pt26set2_F7 | AATATGCCATTGGAGCTTCG |
| Pt26set2_R7 | AGGCAGCTACCGACAAGAAA |
| Pt26set2_F8 | TTGGACCATGGCTCTTATCC |
| Pt26set2_R8 | CCGGCAATACCGATAAAGAA |
| Pt26set2_F9 | CTCCGCCGATAGTAATTGGA |
| Pt26set2_R9 | GAGAAGGACGCGAATCAGAC |
|  | **Primers to amplify KanR-Ars for lambda red insertion** |
| 25red2-F | TGACAAATAAAATGGCCTTACAGTTAGCTCAGAAA GCCAGTGTTACAACCAATTAACC |
| 25red2-R | CGGGCAGTCCACTAACCGTCACTGGCCAATAGTCT GGATCGCTTGCCTGTAACT |
| 25red3-F | CTTGGTGCCCTCTTTTACAACGCCAAGGACGCTAC GCCAGTGTTACAACCAATTAACC |
| 25red3-R | GTTGCGTTGTCATTGGTTGGTGACTGTTTGAGAAG GGATCGCTTGCCTGTAACT |
| 25red4-F | TATTGTGATATCAGTCAATCAAGACTTTAATCATT GCCAGTGTTACAACCAATTAACC |
| 25red4-R2 | GAACCAGGACTTTTTTTGGTCGCATCAGCAATCAG GGATCGCTTGCCTGTAACT |
| 26Red2-F2 | CGATGCCAATTCGTCATCGTACAACGCAACTGTAC GCCAGTGTTACAACCAATTAACC |
| 26Red2-R2 | GATGCGGTGGAGACTTACGTGGTGCTGAACCTTGC GGATCGCTTGCCTGTAACT |
| 26Red3-F3 | TGCATCGGAGAACCCTCGTGAACTGTAAGCGTGATGC GCCAGTGTTACAACCAATTAACC |
| 26Red3-R3 | TACACTTACATTAATAGAGCTCAGGATTCTATCATGTTTC GGATCGCTTGCCTGTAACT |
| 26Red4-F2 | ATTGTCAAGTGATGCAAAAGTGCGAGGTTCCCGCC GCCAGTGTTACAACCAATTAACC |
| 26Red4-R2 | GCGGCTCCGGTCTCGCTAGCTGGATCTTTGCTGCC GGATCGCTTGCCTGTAACT |
|  | **LambdaRed screening primers:** |
| 25-Screen-2F | CCTCAATGAGAGAGCACGACC |
| 25-Screen-2R | CGACTGGTAGTCGGTGTCAAG |
| 25-Screen-3F | TCCGAGTCGAAAGCCCGTTC |
| 25-Screen-3R | GCAGTAGGCGATTGAACTTCT |
| 25-Screen-4F | CCGTGACCCGCCACTCTCAAA |
| 25-Screen-4R | TCGCGCGTGAAGAAGGACTAGG |
| 26-2-screenF | GAATCGTTGTCCAGTCCGCTTG |
| 26-2-screenR | GGGAGCTGTCACAGATGAAGC |
| 26red3checkF3 | CCA CTT CGA GAT TCA CCT GAC |
| 26red3checkR3 | CAA CCA TAC GCA TCG CTT TAC C |
| 26-4-screenF | GTGACCCAGAGTCATCATGACC |
| 26-4-screenR | CTCCGGAATAGTATCCCAGGACG |
|  | **URA insertions** |
| URApt25-3_F | GCATCGACATCAAGGATTTCTATCTTGGGACACCCATGGCTCGCTACGAATACATGCGCATCCCCGTCCC cgttgcaggccatgctgtcc |
| URApt25-3_R | TTTTCGGTGCCGGTGTATTTTATGCCAAAGTTGTCGACAACTAGGGTAAATTGAATCGGGCGTGTGACGT CGATGAAACGAGAGAGGATG |
| URApt26-3_F | TCGGTAACTGAACCACTGAGAGTCCACGTCCTGGGTACCGCCAACAACTGGATCATCCGCCGAAGAAATGcgttgcaggccatgctgtcc |
| URApt26-3_R | AAAAGGGGGTGAATGTGATGAGACGAGCGGTAAATCCAGCAAAAGTTCCAAAAGCGGAAAAGGCAAGGGCCGATGAAACGAGAGAGGATG |
|  | **URA screen** |
| 25-urascreen-F | GCAAGGCAACCTACTTGCGCATG |
| 25-urascreenR2 | GAGTCGACAGCCCTAGCATAA |
| 26-3urascreenR2 | GGGGAGGCAGACAAGGTATAG |
| 26-3urascreenF3 | GTGATTTTTCTCTGGTCCCG |
|  | **Ch25 Assembly vectors primers** |
| Pt25-AV15-1 | AAGTACATCACCGACGAGCAAGGCAAGACGATCTTTTGGATGTGTTCCAAGCGTTGGACA |
| Pt25-AV15-2new | AATGGAGCTATCGCGTCGTTGGGGCATTGGCCTCGAGTCCTGATATTAGCATCCCTACAG |
| Pt25-AV15-3new | GTTGGCAATATTGGCTGTAGGGATGCTAATATCAGGACTCGAGGCCAATGCCCCAACGAC |
| Pt25-AV15-4 | CCCAGCGGCGAGGGCAACCAGTAGAGTTAAATATATGGGCCAGGGGGAAAGAGATAAGAA |
|  | **Ch26 Assembly vector primers** |
| 26-av-15-F1 | GTAAGTACATCACCGACGAGCAAGGCAAGACGATCTGTGTTTCCTCAGGAGGTTAATGT |
| 26-av-15-R1 | TTTAAACCCTGTCGCAAACTTTGTTTTGCGGAACTCGAGGAAACCAAGTTGTAGATCCG |
| 26-av-15-F2 | TTGCAATGGAAGGACGGATCTACAACTTGGTTTCCTCGAGTTCCGCAAAACAAAGTTTG |
| 26-av-15-R2 | GGCCATAGACGGCCGCCAGCCCAGCGGCGAGGGCAACCAGTAGCAAGGGGCTTGCCAGT |
|  | **Primers to check the ends of 25 and 26** |
| Endchk-F | AGGACGAGCTTGGCAAGGT |
| Endchk-R | TTGTCCACATCATCTGTGGGA |
| Pt25-1endchk-R | CATCATCCACAAGCGTAGAGC |
| Pt25-5endchk-F2 | GTGGTCTTCGGTTCTTATCTCTT |
| Pt26-1endchk-R | TGCCGATCCATCCATCTTGT |
| Pt26-5endchk-F2 | TTGTTTGCGACGGATAGCAGC |
|  | **Primers to amplify URA3 knockout cassette to modify fragment 26-3** |
| URA-F | CCAGTAGTGCTCGCCGCAGTCGAGCGACAGGGCGAAGCCCCGTTGCAGGCCATGCTGTCC |
| URA-R100 | TCATAGTCAATGTCAATTCGCGAACAATTCAATCATTGAGCGATGAAACGAGAGAGGATG |
| URA-R90 | TTCGTCAGAATCGTATCCGTTCGAGTCTACTAATAGCCTCCGATGAAACGAGAGAGGATG |
| URA-R80 | GCCACTGGTATGTTGGAGGACCACCTGCAGTCCGTATTGTCGATGAAACGAGAGAGGATG |
| URA-R70 | AGATGGATTGCAATATTTGTGCTGCCTGAACCAGACCTTGCGATGAAACGAGAGAGGATG |
| URA-R60 | TCGATTGTCCTATTTCTTTTCGACCTGGTACGTTTTTGGACGATGAAACGAGAGAGGATG |
| URA-R50 | TTTTGGAAATGAAAATGTGTAAACATTATATGTTGTTGTGCGATGAAACGAGAGAGGATG |
| URA-R40 | AGCATGAGCTCGAGATCGTCGTTGGGACGGCGGACGGAGCCGATGAAACGAGAGAGGATG |
| URA-R30 | TGGTCTGCTCGCCATCTGCCCTCTTTTGGTTGTCAATAATCGATGAAACGAGAGAGGATG |
| URA-R20 | AAGAAATTGCGGGAACTGAGCATGGGAAAGTACGATTGGGCGATGAAACGAGAGAGGATG |
|  | **Primers to confirm the 5’ insertion junction of the URA3 knockout cassette** |
| F-left | AGTGGAGCGGATTATGTCAG |
| R-left | CCTACAATCCATGCCAACCC |
|  | **Primers to confirm the 3’ insertion junction (Forward primer, F-right, common for all fargments)** |
| F-right | CGTGTTTCGTAAAGTCTGGAA |
| 100-R-right | CAAGAAAGAATGATGCCCCTA |
| 90-R-right | CAGTGGGGACGCCATTTCTTCG |
| 80-R-right | CGACATCGCTACGAAGTCAATC |
| 70-R-right | GGTGGAGTGAGGCAATCTCC |
| 60-R-right | GCTATTGTAATACATATAGCAC |
| 50-R-right | GAATTTCCAGTGATGGCGAC |
| 40-R-right | CCAGGCCGAATCGTCTCAC |
| 30-R-right | GGCATCGGTGGCTTGACGTTC |
| 20-R-right | GCACGGCAAAGTAGACCCAG |

**
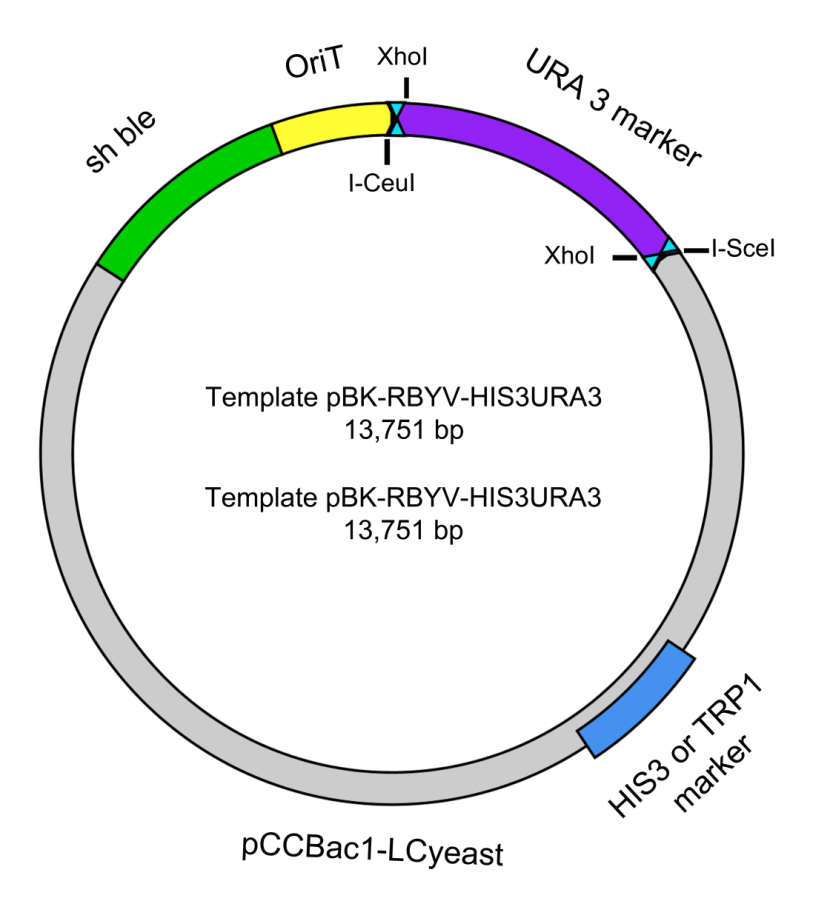
**

**Sequence for pBK-RBYV-HIS3URA3 template vector:**

From 5’ end: URA3 region, XhoI site (red), I-SceI site (red and underlined), pCCBBac1-LCyeast (bold) with HIS3 marker highlighted in blue, ShBle marker (green), OriT (brown) , I-SceI site (red and underlined), XhoI site (red).

cgttgcaggccatgctgtccaggcaggtagatgacgaccatcagggacagcttcaaggatcgctcgcggctcttaccagcctaacttcgatcactggaccgctgatcgtcacggcgatttatgccgcctcggcgagcacatggaacgggttggcatggattgtaggcgccgccctataccttgtctgcctccccgcgttgcgtcgcggtgcatggagccgggccacctcgacctgaatggaagccggcggcacctcgctaacggattcaccactccaagaattggagccaatcaattcttgcggagaactgtgaatgcgcaaaccaacccttggcagaacatatccatcgcgtccgccatctccagcagccgcacgcggcgcatccccccccccctttcaattcaattcatcattttttttttattcttttttttgatttcggtttctttgaaatttttttgattcggtaatctccgaacagaaggaagaacgaaggaaggagcacagacttagattggtatatatacgcatatgtagtgttgaagaaacatgaaattgcccagtattcttaacccaactgcacagaacaaaaacctgcaggaaacgaagataaatcatgtcgaaagctacatataaggaacgtgctgctactcatcctagtcctgttgctgccaagctatttaatatcatgcacgaaaagcaaacaaacttgtgtgcttcattggatgttcgtaccaccaaggaattactggagttagttgaagcattaggtcccaaaatttgtttactaaaaacacatgtggatatcttgactgatttttccatggagggcacagttaagccgctaaaggcattatccgccaagtacaattttttactcttcgaagacagaaaatttgctgacattggtaatacagtcaaattgcagtactctgcgggtgtatacagaatagcagaatgggcagacattacgaatgcacacggtgtggtgggcccaggtattgttagcggtttgaagcaggcggcagaagaagtaacaaaggaacctagaggccttttgatgttagcagaattgtcatgcaagggctccctatctactggagaatatactaagggtactgttgacattgcgaagagcgacaaagattttgttatcggctttattgctcaaagagacatgggtggaagagatgaaggttacgattggttgattatgacacccggtgtgggtttagatgacaagggagacgcattgggtcaacagtatagaaccgtggatgatgtggtctctacaggatctgacattattattgttggaagaggactatttgcaaagggaagggatgctaaggtagagggtgaacgttacagaaaagcaggctgggaagcatatttgagaagatgcggccagcaaaactaaaaaactgtattataagtaaatgcatgtatactaaactcacaaattagagcttcaatttaattatatcagttattactcgggcgtaatgatttttataatgacgaaaaaaaaaaaattggaaagaaaagggggggggggcagcgttgggtcctggccacgggtgcgcatgatcgtgctcctgtcgttgaggacccggctaggctggcggggttgccttactggttagcagaatgaatcaccgatacgcgagcgaacgtgaagcgactgctgctgcaaaacgtctgcgacctgagcaacaacatgaatggtcttcggtttccgtgtttcgtaaagtctggaaacgcggaagtcagcgccctgcaccattatgttccggatctgcatcgcaggatgctgctggctaccctgtggaacacctacatctgtattaacgaagcgctggcattgaccctgagtgatttttctctggtcccgccgcatccataccgccagttgtttaccctcacaacgttccagtaaccgggcatgttcatcatcagtaacccgtatcgtgagcatcctctctcgtttcatcgCTCGAGAGTTACGCTAGGGATAACAGGGTAATATAG**CTGGTTGCCCTCGCCGCTGGGCTGGCGGCCGTCTATGGCCCTGCAAACGCGCCAGAAACGCCGTCGAAGCCGTGTGCGAGACACCGCGGCCGGCCGCCGGCGTTGTGGATACCTCGCGGAAAACTTGGCCCTCACTGACAGATGAGGGGCGGACGTTGACACTTGAGGGGCCGACTCACCCGGCGCGGCGTTGACAGATGAGGGGCAGGCTCGATTTCGGCCGGCGACGTGGAGCTGGCCAGCCTCGCAAATCGGCGAAAACGCCTGATTTTACGCGAGTTTCCCACAGATGATGTGGACAAGCCTGGGGATAAGTGCCCTGCGGTATTGACACTTGAGGGGCGCGACTACTGACAGATGAGGGGCGCGATCCTTGACACTTGAGGGGCAGAGTGCTGACAGATGAGGGGCGCACCTATTGACATTTGAGGGGCTGTCCACAGGCAGAAAATCCAGCATTTGCAAGGGTTTCCGCCCGTTTTTCGGCCACCGCTAACCTGTCTTTTAACCTGCTTTTAAACCAATATTTATAAACCTTGTTTTTAACCAGGGCTGCGCCCTGTGCGCGTGACCGCGCACGCCGAAGGGGGGTGCCCCCCCTTCTCGAACCCTCCCGGTCGAGTGAGCGAGGAAGCACCAGGGAACAGCACTTATATATTCTGCTTACACACGATGCCTGAAAAAACTTCCCTTGGGGTTATCCACTTATCCACGGGGATATTTTTATAATTATTTTTTTTATAGTTTTTAGATCTTCTTTTTTAGAGCGCCTTGTAGGCCTTTATCCATGCTGGTTCTAGAGAAGGTGTTGTGACAAATTGCCCTTTCAGTGTGACAAATCACCCTCAAATGACAGTCCTGTCTGTGACAAATTGCCCTTAACCCTGTGACAAATTGCCCTCAGAAGAAGCTGTTTTTTCACAAAGTTATCCCTGCTTATTGACTCTTTTTTATTTAGTGTGACAATCTAAAAACTTGTCACACTTCACATGGATCTGTCATGGCGGAAACAGCGGTTATCAATCACAAGAAACGTAAAAATAGCCCGCGAATCGTCCAGTCAAACGACCTCACTGAGGCGGCATATAGTCTCTCCCGGGATCAAAAACGTATGCTGTATCTGTTCGTTGACCAGATCAGAAAATCTGATGGCACCCTACAGGAACATGACGGTATCTGCGAGATCCATGTTGCTAAATATGCTGAAATATTCGGATTGACCTCTGCGGAAGCCAGTAAGGATATACGGCAGGCATTGAAGAGTTTCGCGGGGAAGGAAGTGGTTTTTTATCGCCCTGAAGAGGATGCCGGCGATGAAAAAGGCTATGAATCTTTTCCTTGGTTTATCAAACGTGCGCACAGTCCATCCAGAGGGCTTTACAGTGTACATATCAACCCATATCTCATTCCCTTCTTTATCGGGTTACAGAACCGGTTTACGCAGTTTCGGCTTAGTGAAACAAAAGAAATCACCAATCCGTATGCCATGCGTTTATACGAATCCCTGTGTCAGTATCGTAAGCCGGATGGCTCAGGCATCGTCTCTCTGAAAATCGACTGGATCATAGAGCGTTACCAGCTGCCTCAAAGTTACCAGCGTATGCCTGACTTCCGCCGCCGCTTCCTGCAGGTCTGTGTTAATGAGATCAACAGCAGAACTCCAATGCGCCTCTCATACATTGAGAAAAAGAAAGGCCGCCAGACGACTCATATCGTATTTTCCTTCCGCGATATCACTTCCATGACGACAGGATAGTCTGAGGGTTATCTGTCACAGATTTGAGGGTGGTTCGTCACATTTGTTCTGACCTACTGAGGGTAATTTGTCACAGTTTTGCTGTTTCCTTCAGCCTGCATGGATTTTCTCATACTTTTTGAACTGTAATTTTTAAGGAAGCCAAATTTGAGGGCAGTTTGTCACAGTTGATTTCCTTCTCTTTCCCTTCGTCATGTGACCTGATATCGGGGGTTAGTTCGTCATCATTGATGAGGGTTGATTATCACAGTTTATTACTCTGAATTGGCTATCCGCGTGTGTACCTCTACCTGGAGTTTTTCCCACGGTGGATATTTCTTCTTGCGCTGAGCGTAAGAGCTATCTGACAGAACAGTTCTTCTTTGCTTCCTCGCCAGTTCGCTCGCTATGCTCGGTTACACGGCTGCGGCGAGCATCACGTGCTATAAAAATAATTATAATTTAAATTTTTTAATATAAATATATAAATTAAAAATAGAAAGTAAAAAAAGAAATTAAAGAAAAAATAGTTTTTGTTTTCCGAAGATGTAAAAGACTCTAGGGGGATCGCCAACAAATACTACCTTTTATCTTGCTCTTCCTGCTCTCAGGTATTAATGCCGAATTGTTTCATCTTGTCTGTGTAGAAGACCACACACGAAAATCCTGTGATTTTACATTTTACTTATCGTTAATCGAATGTATATCTATTTAATCTGCTTTTCTTGTCTAATAAATATATATGTAAAGTACGCTTTTTGTTGAAATTTTTTAAACCTTTGTTTATTTTTTTTTCTTCATTCCGTAACTCTTCTACCTTCTTTATTTACTTTCTAAAATCCAAATACAAAACATAAAAATAAATAAACACAGAGTAAATTCCCAAATTATTCCATCATTAAAAGATACGAGGCGCGTGTAAGTTACAGGCAAGCGATCCTAGTACACTCTATATTTTTTTATGCCTCGGTAATGATTTTCATTTTTTTTTTTCCACCTAGCGGATGACTCTTTTTTTTTCTTAGCGATTGGCATTATCACATAATGAATTATACATTATATAAAGTAATGTGATTTCTTCGAAGAATATACTAAAAAATGAGCAGGCAAGATAAACGAAGGCAAAGATGACAGAGCAGAAAGCCCTAGTAAAGCGTATTACAAATGAAACCAAGATTCAGATTGCGATCTCTTTAAAGGGTGGTCCCCTAGCGATAGAGCACTCGATCTTCCCAGAAAAAGAGGCAGAAGCAGTAGCAGAACAGGCCACACAATCGCAAGTGATTAACGTCCACACAGGTATAGGGTTTCTGGACCATATGATACATGCTCTGGCCAAGCATTCCGGCTGGTCGCTAATCGTTGAGTGCATTGGTGACTTACACATAGACGACCATCACACCACTGAAGACTGCGGGATTGCTCTCGGTCAAGCTTTTAAAGAGGCCCTACTGGCGCGTGGAGTAAAAAGGTTTGGATCAGGATTTGCGCCTTTGGATGAGGCACTTTCCAGAGCGGTGGTAGATCTTTCGAACAGGCCGTACGCAGTTGTCGAACTTGGTTTGCAAAGGGAGAAAGTAGGAGATCTCTCTTGCGAGATGATCCCGCATTTTCTTGAAAGCTTTGCAGAGGCTAGCAGAATTACCCTCCACGTTGATTGTCTGCGAGGCAAGAATGATCATCACCGTAGTGAGAGTGCGTTCAAGGCTCTTGCGGTTGCCATAAGAGAAGCCACCTCGCCCAATGGTACCAACGATGTTCCCTCCACCAAAGGTGTTCTTATGTAGTTTTACACAGGAGTCTGGACTTGACGCTAGTGATAATAAGTGACTGAGGTATGTGCTCTTCTTATCTCCTTTTGTAGTGTTGCTCTTATTTTAAACAACTTTGCGGTTTTTTGATGACTTTGCGATTTTGTTGTTGCTTTGCAGTAAATTGCAAGATTTAATAAAAAAACGCAAAGCAATGATTAAAGGATGTTCAGAATGAAACTCATGGAAACACTTAACCAGTGCATAAACGCTGGTCATGAAATGACGAAGGCTATCGCCATTGCACAGTTTAATGATGACAGCCCGGAAGCGAGGAAAATAACCCGGCGCTGGAGAATAGGTGAAGCAGCGGATTTAGTTGGGGTTTCTTCTCAGGCTATCAGAGATGCCGAGAAAGCAGGGCGACTACCGCACCCGGATATGGAAATTCGAGGACGGGTTGAGCAACGTGTTGGTTATACAATTGAACAAATTAATCATATGCGTGATGTGTTTGGTACGCGATTGCGACGTGCTGAAGACGTATTTCCACCGGTGATCGGGGTTGCTGCCCATAAAGGTGGCGTTTACAAAACCTCAGTTTCTGTTCATCTTGCTCAGGATCTGGCTCTGAAGGGGCTACGTGTTTTGCTCGTGGAAGGTAACGACCCCCAGGGAACAGCCTCAATGTATCACGGATGGGTACCAGATCTTCATATTCATGCAGAAGACACTCTCCTGCCTTTCTATCTTGGGGAAAAGGACGATGTCACTTATGCAATAAAGCCCACTTGCTGGCCGGGGCTTGACATTATTCCTTCCTGTCTGGCTCTGCACCGTATTGAAACTGAGTTAATGGGCAAATTTGATGAAGGTAAACTGCCCACCGATCCACACCTGATGCTCCGACTGGCCATTGAAACTGTTGCTCATGACTATGATGTCATAGTTATTGACAGCGCGCCTAACCTGGGTATCGGCACGATTAATGTCGTATGTGCTGCTGATGTGCTGATTGTTCCCACGCCTGCTGAGTTGTTTGACTACACCTCCGCACTGCAGTTTTTCGATATGCTTCGTGATCTGCTCAAGAACGTTGATCTTAAAGGGTTCGAGCCTGATGTACGTATTTTGCTTACCAAATACAGCAATAGCAATGGCTCTCAGTCCCCGTGGATGGAGGAGCAAATTCGGGATGCCTGGGGAAGCATGGTTCTAAAAAATGTTGTACGTGAAACGGATGAAGTTGGTAAAGGTCAGATCCGGATGAGAACTGTTTTTGAACAGGCCATTGATCAACGCTCTTCAACTGGTGCCTGGAGAAATGCTCTTTCTATTTGGGAACCTGTCTGCAATGAAATTTTCGATCGTCTGATTAAACCACGCTGGGAGATTAGATAATGAAGCGTGCGCCTGTTATTCCAAAACATACGCTCAATACTCAACCGGTTGAAGATACTTCGTTATCGACACCAGCTGCCCCGATGGTGGATTCGTTAATTGCGCGCGTAGGAGTAATGGCTCGCGGTAATGCCATTACTTTGCCTGTATGTGGTCGGGATGTGAAGTTTACTCTTGAAGTGCTCCGGGGTGATAGTGTTGAGAAGACCTCTCGGGTATGGTCAGGTAATGAACGTGACCAGGAGCTGCTTACTGAGGACGCACTGGATGATCTCATCCCTTCTTTTCTACTGACTGGTCAACAGACACCGGCGTTCGGTCGAAGAGTATCTGGTGTCATAGAAATTGCCGATGGGAGTCGCCGTCGTAAAGCTGCTGCACTTACCGAAAGTGATTATCGTGTTCTGGTTGGCGAGCTGGATGATGAGCAGATGGCTGCATTATCCAGATTGGGTAACGATTATCGCCCAACAAGTGCTTATGAACGTGGTCAGCGTTATGCAAGCCGATTGCAGAATGAATTTGCTGGAAATATTTCTGCGCTGGCTGATGCGGAAAATATTTCACGTAAGATTATTACCCGCTGTATCAACACCGCCAAATTGCCTAAATCAGTTGTTGCTCTTTTTTCTCACCCCGGTGAACTATCTGCCCGGTCAGGTGATGCACTTCAAAAAGCCTTTACAGATAAAGAGGAATTACTTAAGCAGCAGGCATCTAACCTTCATGAGCAGAAAAAAGCTGGGGTGATATTTGAAGCTGAAGAAGTTATCACTCTTTTAACTTCTGTGCTTAAAACGTCATCTGCATCAAGAACTAGTTTAAGCTCACGACATCAGTTTGCTCCTGGAGCGACAGTATTGTATAAGGGCGATAAAATGGTGCTTAACCTGGACAGGTCTCGTGTTCCAACTGAGTGTATAGAGAAAATTGAGGCCATTCTTAAGGAACTTGAAAAGCCAGCACCCTGATGCGACCACGTTTTAGTCTACGTTTATCTGTCTTTACTTAATGTCCTTTGTTACAGGCCAGAAAGCATAACTGGCCTGAATATTCTCTCTGGGCCCACTGTTCCACTTGTATCGTCGGTCTGATAATCAGACTGGGACCACGGTCCCACTCGTATCGTCGGTCTGATTATTAGTCTGGGACCACGGTCCCACTCGTATCGTCGGTCTGATTATTAGTCTGGGACCACGGTCCCACTCGTATCGTCGGTCTGATAATCAGACTGGGACCACGGTCCCACTCGTATCGTCGGTCTGATTATTAGTCTGGGACCATGGTCCCACTCGTATCGTCGGTCTGATTATTAGTCTGGGACCACGGTCCCACTCGTATCGTCGGTCTGATTATTAGTCTGGAACCACGGTCCCACTCGTATCGTCGGTCTGATTATTAGTCTGGGACCACGGTCCCACTCGTATCGTCGGTCTGATTATTAGTCTGGGACCACGATCCCACTCGTGTTGTCGGTCTGATTATCGGTCTGGGACCACGGTCCCACTTGTATTGTCGATCAGACTATCAGCGTGAGACTACGATTCCATCAATGCCTGTCAAGGGCAAGTATTGACATGTCGTCGTAACCTGTAGAACGGAGTAACCTCGGTGTGCGGTTGTATGCCTGCTGTGGATTGCTGCTGTGTCCTGCTTATCCACAACATTTTGCGCACGGTTATGTGGACAAAATACCTGGTTACCCAGGCCGTGCCGGCACGTTAACCGGGCTGCATCCGATGCAAGTGTGTCGCTGTCGACGAGCTCGCGAGCTCGGACATGAGGTTGCCCCGTATTCAGTGTCGCTGATTTGTATTGTCTGAAGTTGTTTTTACGTTAAGTTGATGCAGATCAATTAATACGATACCTGCGTCATAATTGATTATTTGACGTGGTTTGATGGCCTCCACGCACGTTGTGATATGTAGATGATAATCATTATCACTTTACGGGTCCTTTCCGGTGATCCGACAGGTTACGGGGCGGCGACCTCGCGGGTTTTCGCTATTTATGAAAATTTTCCGGTTTAAGGCGTTTCCGTTCTTCTTCGTCATAACTTAATGTTTTTATTTAAAATACCCTCTGAAAAGAAAGGAAACGACAGGTGCTGAAAGCGAGCTTTTTGGCCTCTGTCGTTTCCTTTCTCTGTTTTTGTCCGTGGAATGAACAATGGAAGTCCGAGCTCATCGCTAATAACTTCGTATAGCATACATTATACGAAGTTATATTCGATGCGGCCGCAAGGGGTTCGCGTCAGCGGGTGTTGGCGGGTGTCGGGGCTGGCTTAACTATGCGGCATCAGAGCAGATTGTACTGAGAGTGCACCATATGCGGTGTGAAATACCACACAGATGCGTAAGGAGAAAATACCGCATCAGGCGCCATTCGCCATTCAGCTGCGCAACTGTTGGGAAGGGCGATCGGTGCGGGCCTCTTCGCTATTACGCCAGCTGGCGAAAGGGGGATGTGCTGCAAGGCGATTAAGTTGGGTAACGCCAGGGTTTTCCCAGTCACGACGTTGTAAAACGACGGCCAGTGAATTGTAATACGACTCACTATAGGGCGAATTCGAGCTCGGTACCCGGGGATCCTCTAGAGTCGACCTGCAGGCATGCAAGCTTGAGTATTCTATAGTCTCACCTAAATAGCTTGGCGTAATCATGGTCATAGCTGTTTCCTGTGTGAAATTGTTATCCGCTCACAATTCCACACAACATACGAGCCGGAAGCATAAAGTGTAAAGCCTGGGGTGCCTAATGAGTGAGCTAACTCACATTAATTGCGTTGCGCTCACTGCCCGCTTTCCAGTCGGGAAACCTGTCGTGCCAGCTGCATTAATGAATCGGCCAACGCGAACCCCTTGCGGCCGCCCGGGCCGTCGACCAATTCTCATGTTTGACAGCTTATCATCGAATTTCTGCCATTCATCCGCTTATTATCACTTATTCAGGCGTAGCAACCAGGCGTTTAAGGGCACCAATAACTGCCTTAAAAAAATTACGCCCCGCCCTGCCACTCATCGCAGTACTGTTGTAATTCATTAAGCATTCTGCCGACATGGAAGCCATCACAAACGGCATGATGAACCTGAATCGCCAGCGGCATCAGCACCTTGTCGCCTTGCGTATAATATTTGCCCATGGTGAAAACGGGGGCGAAGAAGTTGTCCATATTGGCCACGTTTAAATCAAAACTGGTGAAACTCACCCAGGGATTGGCTGAGACGAAAAACATATTCTCAATAAACCCTTTAGGGAAATAGGCCAGGTTTTCACCGTAACACGCCACATCTTGCGAATATATGTGTAGAAACTGCCGGAAATCGTCGTGGTATTCACTCCAGAGCGATGAAAACGTTTCAGTTTGCTCATGGAAAACGGTGTAACAAGGGTGAACACTATCCCATATCACCAGCTCACCGTCTTTCATTGCCATACGAAATTCCGGATGAGCATTCATCAGGCGGGCAAGAATGTGAATAAAGGCCGGATAAAACTTGTGCTTATTTTTCTTTACGGTCTTTAAAAAGGCCGTAATATCCAGCTGAACGGTCTGGTTATAGGTACATTGAGCAACTGACTGAAATGCCTCAAAATGTTCTTTACGATGCCATTGGGATATATCAACGGTGGTATATCCAGTGATTTTTTTCTCCATTTTAGCTTCCTTAGCTCCTGAAAATCTCGATAACTCAAAAAATACGCCCGGTAGTGATCTTATTTCATTATGGTGAAAGTTGGAACCTCTTACGTGCCGATCAACGTCTCATTTTCGCCAAAAGTTGGCCCAGGGCTTCCCGGTATCAACAGGGACACCAGGATTTATTTATTCTGCGAAGTGATCTTCCGTCACAGGTATTTATTCGCGATAAGCTCATGGAGCGGCGTAACCGTCGCACAGGAAGGACAGAGAAAGCGCGGATCTGGGAAGTGACGGACAGAACGGTCAGGACCTGGATTGGGGAGGCGGTTGCCGCCGCTGCTGCTGACGGTGTGACGTTCTCTGTTCCGGTCACACCACATACGTTCCGCCATTCCTATGCGATGCACATGCTGTATGCCGGTATACCGCTGAAAGTTCTGCAAAGCCTGATGGGACATAAGTCCATCAGTTCAACGGAAGTCTACACGAAGGTTTTTGCGCTGGATGTGGCTGCCCGGCACCGGGTGCAGTTTGCGATGCCGGAGTCTGATGCGGTTGCGATGCTGAAACAATTATCCTGAGAATAAATGCCTTGGCCTTTATATGGAAATGTGGAACTGAGTGGATATGCTGTTTTTGTCTGTTAAACAGAGAAGCTGGCTGTTATCCACTGAGAAGCGAACGAAACAGTCGGGAAAATCTCCCATTATCGTAGAGATCCGCATTATTAATCTCAGGAGCCTGTGTAGCGTTTATAGGAAGTAGTGTTCTGTCATGATGCCTGCAAGCGGTAACGAAAACGATTTGAATATGCCTTCAGGAACAATAGAAATCTTCGTGCGGTGTTACGTTGAAGTGGAGCGGATTATGTCAGCAATGGACAGAACAACCTAATGAACACAGAACCATGATGTGGTCTGTCCTTTTACAGCCAGTAGTGCTCGCCGCAGTCGAGCGACAGGGCGAAGCCC**ACCATGATTACGCCAAGCTCGAAATTAACCCTCACTAAAGGGAACAAAAGCTGGTACCTAACAGGATTAGTGCAATTCGAGTTGAATCACTGGGAAAAACATTGTCTTCTTTTTTATATTATCATTTGCATTAGTGCTGCAGTCGTAGATACTTGTTGGTTGAAAGACATCAGCTGGGAGGGACTGGACTAGCGTTTGGTAAGGAGACATACCTGTTAACGTTGGTTGCAAAATTCCATTTCGCGATTTATGTTATCTGTAAATCCTGATTTGTCTGGAATTCTTGATACTTCCGTTTTTTTAGAGGCCAATGATTAGCATCGGCGATTCTCAAAATAGCATTTTCGACATGCGGTGCTGATTTCATAAACATAGACAACGCTTTTACATGTAAAAGTAACTTGCGGACTTGGAACAGTGCTCTGTTTTTGGTGTGAACGTAACTCAGCAATATTTCTGTGCTAGCAAGGTTTTTTATGATCGACCGAAGATCTCAAAACTCCGGGTCTTTCAACTGTCTGACTAGACCATGTTCGTAACGTCGGACAGCAGCTTTCGTTGTACTGGTAGAATTTCTACGTGCGAAGCACGTGTAGGCAGGTTGAACGACGATCCCTGCCGATGGATGGATTGGCACGCGGCGGAACGCTTTCGTGATCTACACCACCTGGATCTTCACATATCTTCGAAATCGAAAAATTAACCAAGTCGACGGTATCGATAATATTCTAGCTGAGGGTACCCATGGCCAAGTTGACCAGTGCCGTTCCGGTGCTCACCGCGCGCGACGTCGCCGGAGCGGTCGAGTTCTGGACCGACCGGCTCGGGTTCTCCCGGGACTTCGTGGAGGACGACTTCGCCGGTGTGGTCCGGGACGACGTGACCCTGTTCATCAGCGCGGTCCAGGACCAGGTGGTGCCGGACAACACCCTGGCCTGGGTGTGGGTGCGCGGCCTGGACGAGCTGTACGCCGAGTGGTCGGAGGTCGTGTCCACGAACTTCCGGGACGCCTCCGGGCCGGCCATGACCGAGATCGGCGAGCAGCCGTGGGGGCGGGAGTTCGCCCTGCGCGACCCGGCCGGCAACTGCGTGCACTTCGTGGCCGAGGAGCAGGACTGACCGACGCCGACCAACACCGCCGGTCCGACGCGGCCCGACGGGTCCGAGGCCTCGGAGATCTGGGCCCATGCGGCCGCAACAACTACCTCGACTTTGGCTGGGACACTTTCAGTGAGGACAAGAAGCTTCAGAAGCGTGCTATCGAACTCAACCAGGGACGTGCGGCACAAATGGGCATCCTTGCTCTCATGGTGCACGAACAGTTGGGAGTCTCTATCCTTCCTTAAAAATTTAATTTTCATTAGTTGCAGTCACTCCGCTTTGGTTTCACAGTCAGGAATAACACTAGCTCGTCTTCACATCTTCCGCTGCATAACCCTGCTTCGGGGTCATTATAGCGATTTTTTCGGTATATCCATCCTTTTTCGCACGATATACAGGATTTTGCCAAAGGGTTCGCGTAGACTTTCCTTGGTGTATCCAACGGCGTCAGCCGGGCAGGATAGGTGAAGTAGGCCCACCCGCGAGCGGGTGTTCCTTCTTCACTGTCCCTTATTCGCACCTGGCGGTGCTCAACGGGAATCCTGCTCTGCGAGGCTGGCCGGCTACCGCCGGCGTAACAGATGAGGGCAAGCGGATGGCTGATGAAACCAAGCCAACCAGGAAGGGCAGCCCACCTATCAAGGTGTACTGCCTTCCAGACGAACGAAGAGCGATTGAGGAAAAGGCGGCGGCGGCCGGCATGAGCCTGTCGGCCTACCTGCTGGCCGTCGGCCAGGGCTACAAAATCACGGGCGTCGTGGACTATGAGCACGTCCGCGAGCTGGCCCGCATCAATGGCGACCTGGGCCGCCTGGGCGGCCTGCTGAAACTCTGGCTCACCGACGACCCGCGCACGGCGCGGTTCGGTGATGCCACGATCCTCGCCCTGCTGGCGAAGATCGAAGAGAAGCAGGACGAGCTTGGCAAGGTCATGATGGGCGTGGTCCGCCCGAGGGCAGAGCCATGACTTTTTTCACGTAACGGATCGGGGTGCGCGTGATTCGGAAGCACGTTCCATGGCCTCCATCAAGAAGAGGCACTTCGAGCTGTAAGTACATCACCGACGAGCAAGGCAAGACGATCCGTAACTATAACGGTCCTAAGGTAGCGAACTCGAG

**Sequence for pBK-RBYV-HIS3URA3 template vector:**

From 5’ end: URA3 region, XhoI site (red), I-sceI site (red and underlined), pCCBBac1-LCyeast (bold) with TRP1 marker highlighted in blue, shble marker (green), OriT (brown), I-sceI site (red and underlined), XhoI site (red).

cgttgcaggccatgctgtccaggcaggtagatgacgaccatcagggacagcttcaaggatcgctcgcggctcttaccagcctaacttcgatcactggaccgctgatcgtcacggcgatttatgccgcctcggcgagcacatggaacgggttggcatggattgtaggcgccgccctataccttgtctgcctccccgcgttgcgtcgcggtgcatggagccgggccacctcgacctgaatggaagccggcggcacctcgctaacggattcaccactccaagaattggagccaatcaattcttgcggagaactgtgaatgcgcaaaccaacccttggcagaacatatccatcgcgtccgccatctccagcagccgcacgcggcgcatccccccccccctttcaattcaattcatcattttttttttattcttttttttgatttcggtttctttgaaatttttttgattcggtaatctccgaacagaaggaagaacgaaggaaggagcacagacttagattggtatatatacgcatatgtagtgttgaagaaacatgaaattgcccagtattcttaacccaactgcacagaacaaaaacctgcaggaaacgaagataaatcatgtcgaaagctacatataaggaacgtgctgctactcatcctagtcctgttgctgccaagctatttaatatcatgcacgaaaagcaaacaaacttgtgtgcttcattggatgttcgtaccaccaaggaattactggagttagttgaagcattaggtcccaaaatttgtttactaaaaacacatgtggatatcttgactgatttttccatggagggcacagttaagccgctaaaggcattatccgccaagtacaattttttactcttcgaagacagaaaatttgctgacattggtaatacagtcaaattgcagtactctgcgggtgtatacagaatagcagaatgggcagacattacgaatgcacacggtgtggtgggcccaggtattgttagcggtttgaagcaggcggcagaagaagtaacaaaggaacctagaggccttttgatgttagcagaattgtcatgcaagggctccctatctactggagaatatactaagggtactgttgacattgcgaagagcgacaaagattttgttatcggctttattgctcaaagagacatgggtggaagagatgaaggttacgattggttgattatgacacccggtgtgggtttagatgacaagggagacgcattgggtcaacagtatagaaccgtggatgatgtggtctctacaggatctgacattattattgttggaagaggactatttgcaaagggaagggatgctaaggtagagggtgaacgttacagaaaagcaggctgggaagcatatttgagaagatgcggccagcaaaactaaaaaactgtattataagtaaatgcatgtatactaaactcacaaattagagcttcaatttaattatatcagttattactcgggcgtaatgatttttataatgacgaaaaaaaaaaaattggaaagaaaagggggggggggcagcgttgggtcctggccacgggtgcgcatgatcgtgctcctgtcgttgaggacccggctaggctggcggggttgccttactggttagcagaatgaatcaccgatacgcgagcgaacgtgaagcgactgctgctgcaaaacgtctgcgacctgagcaacaacatgaatggtcttcggtttccgtgtttcgtaaagtctggaaacgcggaagtcagcgccctgcaccattatgttccggatctgcatcgcaggatgctgctggctaccctgtggaacacctacatctgtattaacgaagcgctggcattgaccctgagtgatttttctctggtcccgccgcatccataccgccagttgtttaccctcacaacgttccagtaaccgggcatgttcatcatcagtaacccgtatcgtgagcatcctctctcgtttcatcgCTCGAGAGTTACGCTAGGGATAACAGGGTAATATAG**CTGGTTGCCCTCGCCGCTGGGCTGGCGGCCGTCTATGGCCCTGCAAACGCGCCAGAAACGCCGTCGAAGCCGTGTGCGAGACACCGCGGCCGGCCGCCGGCGTTGTGGATACCTCGCGGAAAACTTGGCCCTCACTGACAGATGAGGGGCGGACGTTGACACTTGAGGGGCCGACTCACCCGGCGCGGCGTTGACAGATGAGGGGCAGGCTCGATTTCGGCCGGCGACGTGGAGCTGGCCAGCCTCGCAAATCGGCGAAAACGCCTGATTTTACGCGAGTTTCCCACAGATGATGTGGACAAGCCTGGGGATAAGTGCCCTGCGGTATTGACACTTGAGGGGCGCGACTACTGACAGATGAGGGGCGCGATCCTTGACACTTGAGGGGCAGAGTGCTGACAGATGAGGGGCGCACCTATTGACATTTGAGGGGCTGTCCACAGGCAGAAAATCCAGCATTTGCAAGGGTTTCCGCCCGTTTTTCGGCCACCGCTAACCTGTCTTTTAACCTGCTTTTAAACCAATATTTATAAACCTTGTTTTTAACCAGGGCTGCGCCCTGTGCGCGTGACCGCGCACGCCGAAGGGGGGTGCCCCCCCTTCTCGAACCCTCCCGGTCGAGTGAGCGAGGAAGCACCAGGGAACAGCACTTATATATTCTGCTTACACACGATGCCTGAAAAAACTTCCCTTGGGGTTATCCACTTATCCACGGGGATATTTTTATAATTATTTTTTTTATAGTTTTTAGATCTTCTTTTTTAGAGCGCCTTGTAGGCCTTTATCCATGCTGGTTCTAGAGAAGGTGTTGTGACAAATTGCCCTTTCAGTGTGACAAATCACCCTCAAATGACAGTCCTGTCTGTGACAAATTGCCCTTAACCCTGTGACAAATTGCCCTCAGAAGAAGCTGTTTTTTCACAAAGTTATCCCTGCTTATTGACTCTTTTTTATTTAGTGTGACAATCTAAAAACTTGTCACACTTCACATGGATCTGTCATGGCGGAAACAGCGGTTATCAATCACAAGAAACGTAAAAATAGCCCGCGAATCGTCCAGTCAAACGACCTCACTGAGGCGGCATATAGTCTCTCCCGGGATCAAAAACGTATGCTGTATCTGTTCGTTGACCAGATCAGAAAATCTGATGGCACCCTACAGGAACATGACGGTATCTGCGAGATCCATGTTGCTAAATATGCTGAAATATTCGGATTGACCTCTGCGGAAGCCAGTAAGGATATACGGCAGGCATTGAAGAGTTTCGCGGGGAAGGAAGTGGTTTTTTATCGCCCTGAAGAGGATGCCGGCGATGAAAAAGGCTATGAATCTTTTCCTTGGTTTATCAAACGTGCGCACAGTCCATCCAGAGGGCTTTACAGTGTACATATCAACCCATATCTCATTCCCTTCTTTATCGGGTTACAGAACCGGTTTACGCAGTTTCGGCTTAGTGAAACAAAAGAAATCACCAATCCGTATGCCATGCGTTTATACGAATCCCTGTGTCAGTATCGTAAGCCGGATGGCTCAGGCATCGTCTCTCTGAAAATCGACTGGATCATAGAGCGTTACCAGCTGCCTCAAAGTTACCAGCGTATGCCTGACTTCCGCCGCCGCTTCCTGCAGGTCTGTGTTAATGAGATCAACAGCAGAACTCCAATGCGCCTCTCATACATTGAGAAAAAGAAAGGCCGCCAGACGACTCATATCGTATTTTCCTTCCGCGATATCACTTCCATGACGACAGGATAGTCTGAGGGTTATCTGTCACAGATTTGAGGGTGGTTCGTCACATTTGTTCTGACCTACTGAGGGTAATTTGTCACAGTTTTGCTGTTTCCTTCAGCCTGCATGGATTTTCTCATACTTTTTGAACTGTAATTTTTAAGGAAGCCAAATTTGAGGGCAGTTTGTCACAGTTGATTTCCTTCTCTTTCCCTTCGTCATGTGACCTGATATCGGGGGTTAGTTCGTCATCATTGATGAGGGTTGATTATCACAGTTTATTACTCTGAATTGGCTATCCGCGTGTGTACCTCTACCTGGAGTTTTTCCCACGGTGGATATTTCTTCTTGCGCTGAGCGTAAGAGCTATCTGACAGAACAGTTCTTCTTTGCTTCCTCGCCAGTTCGCTCGCTATGCTCGGTTACACGGCTGCGGCGAGCATCACGTGCTATAAAAATAATTATAATTTAAATTTTTTAATATAAATATATAAATTAAAAATAGAAAGTAAAAAAAGAAATTAAAGAAAAAATAGTTTTTGTTTTCCGAAGATGTAAAAGACTCTAGGGGGATCGCCAACAAATACTACCTTTTATCTTGCTCTTCCTGCTCTCAGGTATTAATGCCGAATTGTTTCATCTTGTCTGTGTAGAAGACCACACACGAAAATCCTGTGATTTTACATTTTACTTATCGTTAATCGAATGTATATCTATTTAATCTGCTTTTCTTGTCTAATAAATATATATGTAAAGTACGCTTTTTGTTGAAATTTTTTAAACCTTTGTTTATTTTTTTTTCTTCATTCCGTAACTCTTCTACCTTCTTTATTTACTTTCTAAAATCCAAATACAAAACATAAAAATAAATAAACACAGAGTAAATTCCCAAATTATTCCATCATTAAAAGATACGAGGCGCGTGTAAGTTACAGGCAAGCGATCCAGATGGCAGTAGTGGAAGATATTCTTTATTGAAAAATAGCTTGTCACCTTACGTACAATCTTGATCCGGAGCTTTTCTTTTTTTGCCGATTAAGAATTAATTCGGTCGAAAAAAGAAAAGGAGAGGGCCAAGAGGGAGGGCATTGGTGACTATTGAGCACGTGAGTATACGTGATTAAGCACACAAAGGCAGCTTGGAGTATGTCTGTTATTAATTTCACAGGTAGTTCTGGTCCATTGGTGAAAGTTTGCGGCTTGCAGAGCACAGAGGCCGCAGAATGTGCTCTAGATTCCGATGCTGACTTGCTGGGTATTATATGTGTGCCCAATAGAAAGAGAACAATTGACCCGGTTATTGCAAGGAAAATTTCAAGTCTTGTAAAAGCATATAAAAATAGTTCAGGCACTCCGAAATACTTGGTTGGCGTGTTTCGTAATCAACCTAAGGAGGATGTTTTGGCTCTGGTCAATGATTACGGCATTGATATCGTCCAACTGCATGGAGATGAGTCGTGGCAAGAATACCAAGAGTTCCTCGGTTTGCCAGTTATTAAAAGACTCGTATTTCCAAAAGACTGCAACATACTACTCAGTGCAGCTTCACAGAAACCTCATTCGTTTATTCCCTTGTTTGATTCAGAAGCAGGTGGGACAGGTGAACTTTTGGATTGGAACTCGATTTCTGACTGGGTTGGAAGGCAAGAGAGCCCCGAAAGCTTACATTTTATGTTAGCTGGTGGACTGACGCCAGAAAATGTTGGTGATGCGCTTAGATTAAATGGCGTTATTGGTGTTGATGTAAGCGGAGGTGTGGAGACAAATGGTGTAAAAGACTCTAACAAAATAGCAAATTTCGTCAAAAATGCTAAGAAATAGGTTATTACTGAGTAGTATTTATTTAAGTATTGTTTGTGCACTTGCCTGCGGTGTGAAATACCGCACAGATGCGTAAGGAGAAAATACCGCATCAGGAAATTGTAAACGTTAATATTTTGTTAAAATTCGCGTTAAATTTTTGTTAAATCAGCTCATTTTTTAACCAATAGGCCGAAATCGGCAAAATCCCTTATAAATCATTTTACACAGGAGTCTGGACTTGACGCTAGTGATAATAAGTGACTGAGGTATGTGCTCTTCTTATCTCCTTTTGTAGTGTTGCTCTTATTTTAAACAACTTTGCGGTTTTTTGATGACTTTGCGATTTTGTTGTTGCTTTGCAGTAAATTGCAAGATTTAATAAAAAAACGCAAAGCAATGATTAAAGGATGTTCAGAATGAAACTCATGGAAACACTTAACCAGTGCATAAACGCTGGTCATGAAATGACGAAGGCTATCGCCATTGCACAGTTTAATGATGACAGCCCGGAAGCGAGGAAAATAACCCGGCGCTGGAGAATAGGTGAAGCAGCGGATTTAGTTGGGGTTTCTTCTCAGGCTATCAGAGATGCCGAGAAAGCAGGGCGACTACCGCACCCGGATATGGAAATTCGAGGACGGGTTGAGCAACGTGTTGGTTATACAATTGAACAAATTAATCATATGCGTGATGTGTTTGGTACGCGATTGCGACGTGCTGAAGACGTATTTCCACCGGTGATCGGGGTTGCTGCCCATAAAGGTGGCGTTTACAAAACCTCAGTTTCTGTTCATCTTGCTCAGGATCTGGCTCTGAAGGGGCTACGTGTTTTGCTCGTGGAAGGTAACGACCCCCAGGGAACAGCCTCAATGTATCACGGATGGGTACCAGATCTTCATATTCATGCAGAAGACACTCTCCTGCCTTTCTATCTTGGGGAAAAGGACGATGTCACTTATGCAATAAAGCCCACTTGCTGGCCGGGGCTTGACATTATTCCTTCCTGTCTGGCTCTGCACCGTATTGAAACTGAGTTAATGGGCAAATTTGATGAAGGTAAACTGCCCACCGATCCACACCTGATGCTCCGACTGGCCATTGAAACTGTTGCTCATGACTATGATGTCATAGTTATTGACAGCGCGCCTAACCTGGGTATCGGCACGATTAATGTCGTATGTGCTGCTGATGTGCTGATTGTTCCCACGCCTGCTGAGTTGTTTGACTACACCTCCGCACTGCAGTTTTTCGATATGCTTCGTGATCTGCTCAAGAACGTTGATCTTAAAGGGTTCGAGCCTGATGTACGTATTTTGCTTACCAAATACAGCAATAGCAATGGCTCTCAGTCCCCGTGGATGGAGGAGCAAATTCGGGATGCCTGGGGAAGCATGGTTCTAAAAAATGTTGTACGTGAAACGGATGAAGTTGGTAAAGGTCAGATCCGGATGAGAACTGTTTTTGAACAGGCCATTGATCAACGCTCTTCAACTGGTGCCTGGAGAAATGCTCTTTCTATTTGGGAACCTGTCTGCAATGAAATTTTCGATCGTCTGATTAAACCACGCTGGGAGATTAGATAATGAAGCGTGCGCCTGTTATTCCAAAACATACGCTCAATACTCAACCGGTTGAAGATACTTCGTTATCGACACCAGCTGCCCCGATGGTGGATTCGTTAATTGCGCGCGTAGGAGTAATGGCTCGCGGTAATGCCATTACTTTGCCTGTATGTGGTCGGGATGTGAAGTTTACTCTTGAAGTGCTCCGGGGTGATAGTGTTGAGAAGACCTCTCGGGTATGGTCAGGTAATGAACGTGACCAGGAGCTGCTTACTGAGGACGCACTGGATGATCTCATCCCTTCTTTTCTACTGACTGGTCAACAGACACCGGCGTTCGGTCGAAGAGTATCTGGTGTCATAGAAATTGCCGATGGGAGTCGCCGTCGTAAAGCTGCTGCACTTACCGAAAGTGATTATCGTGTTCTGGTTGGCGAGCTGGATGATGAGCAGATGGCTGCATTATCCAGATTGGGTAACGATTATCGCCCAACAAGTGCTTATGAACGTGGTCAGCGTTATGCAAGCCGATTGCAGAATGAATTTGCTGGAAATATTTCTGCGCTGGCTGATGCGGAAAATATTTCACGTAAGATTATTACCCGCTGTATCAACACCGCCAAATTGCCTAAATCAGTTGTTGCTCTTTTTTCTCACCCCGGTGAACTATCTGCCCGGTCAGGTGATGCACTTCAAAAAGCCTTTACAGATAAAGAGGAATTACTTAAGCAGCAGGCATCTAACCTTCATGAGCAGAAAAAAGCTGGGGTGATATTTGAAGCTGAAGAAGTTATCACTCTTTTAACTTCTGTGCTTAAAACGTCATCTGCATCAAGAACTAGTTTAAGCTCACGACATCAGTTTGCTCCTGGAGCGACAGTATTGTATAAGGGCGATAAAATGGTGCTTAACCTGGACAGGTCTCGTGTTCCAACTGAGTGTATAGAGAAAATTGAGGCCATTCTTAAGGAACTTGAAAAGCCAGCACCCTGATGCGACCACGTTTTAGTCTACGTTTATCTGTCTTTACTTAATGTCCTTTGTTACAGGCCAGAAAGCATAACTGGCCTGAATATTCTCTCTGGGCCCACTGTTCCACTTGTATCGTCGGTCTGATAATCAGACTGGGACCACGGTCCCACTCGTATCGTCGGTCTGATTATTAGTCTGGGACCACGGTCCCACTCGTATCGTCGGTCTGATTATTAGTCTGGGACCACGGTCCCACTCGTATCGTCGGTCTGATAATCAGACTGGGACCACGGTCCCACTCGTATCGTCGGTCTGATTATTAGTCTGGGACCATGGTCCCACTCGTATCGTCGGTCTGATTATTAGTCTGGGACCACGGTCCCACTCGTATCGTCGGTCTGATTATTAGTCTGGAACCACGGTCCCACTCGTATCGTCGGTCTGATTATTAGTCTGGGACCACGGTCCCACTCGTATCGTCGGTCTGATTATTAGTCTGGGACCACGATCCCACTCGTGTTGTCGGTCTGATTATCGGTCTGGGACCACGGTCCCACTTGTATTGTCGATCAGACTATCAGCGTGAGACTACGATTCCATCAATGCCTGTCAAGGGCAAGTATTGACATGTCGTCGTAACCTGTAGAACGGAGTAACCTCGGTGTGCGGTTGTATGCCTGCTGTGGATTGCTGCTGTGTCCTGCTTATCCACAACATTTTGCGCACGGTTATGTGGACAAAATACCTGGTTACCCAGGCCGTGCCGGCACGTTAACCGGGCTGCATCCGATGCAAGTGTGTCGCTGTCGACGAGCTCGCGAGCTCGGACATGAGGTTGCCCCGTATTCAGTGTCGCTGATTTGTATTGTCTGAAGTTGTTTTTACGTTAAGTTGATGCAGATCAATTAATACGATACCTGCGTCATAATTGATTATTTGACGTGGTTTGATGGCCTCCACGCACGTTGTGATATGTAGATGATAATCATTATCACTTTACGGGTCCTTTCCGGTGATCCGACAGGTTACGGGGCGGCGACCTCGCGGGTTTTCGCTATTTATGAAAATTTTCCGGTTTAAGGCGTTTCCGTTCTTCTTCGTCATAACTTAATGTTTTTATTTAAAATACCCTCTGAAAAGAAAGGAAACGACAGGTGCTGAAAGCGAGCTTTTTGGCCTCTGTCGTTTCCTTTCTCTGTTTTTGTCCGTGGAATGAACAATGGAAGTCCGAGCTCATCGCTAATAACTTCGTATAGCATACATTATACGAAGTTATATTCGATGCGGCCGCAAGGGGTTCGCGTCAGCGGGTGTTGGCGGGTGTCGGGGCTGGCTTAACTATGCGGCATCAGAGCAGATTGTACTGAGAGTGCACCATATGCGGTGTGAAATACCACACAGATGCGTAAGGAGAAAATACCGCATCAGGCGCCATTCGCCATTCAGCTGCGCAACTGTTGGGAAGGGCGATCGGTGCGGGCCTCTTCGCTATTACGCCAGCTGGCGAAAGGGGGATGTGCTGCAAGGCGATTAAGTTGGGTAACGCCAGGGTTTTCCCAGTCACGACGTTGTAAAACGACGGCCAGTGAATTGTAATACGACTCACTATAGGGCGAATTCGAGCTCGGTACCCGGGGATCCTCTAGAGTCGACCTGCAGGCATGCAAGCTTGAGTATTCTATAGTCTCACCTAAATAGCTTGGCGTAATCATGGTCATAGCTGTTTCCTGTGTGAAATTGTTATCCGCTCACAATTCCACACAACATACGAGCCGGAAGCATAAAGTGTAAAGCCTGGGGTGCCTAATGAGTGAGCTAACTCACATTAATTGCGTTGCGCTCACTGCCCGCTTTCCAGTCGGGAAACCTGTCGTGCCAGCTGCATTAATGAATCGGCCAACGCGAACCCCTTGCGGCCGCCCGGGCCGTCGACCAATTCTCATGTTTGACAGCTTATCATCGAATTTCTGCCATTCATCCGCTTATTATCACTTATTCAGGCGTAGCAACCAGGCGTTTAAGGGCACCAATAACTGCCTTAAAAAAATTACGCCCCGCCCTGCCACTCATCGCAGTACTGTTGTAATTCATTAAGCATTCTGCCGACATGGAAGCCATCACAAACGGCATGATGAACCTGAATCGCCAGCGGCATCAGCACCTTGTCGCCTTGCGTATAATATTTGCCCATGGTGAAAACGGGGGCGAAGAAGTTGTCCATATTGGCCACGTTTAAATCAAAACTGGTGAAACTCACCCAGGGATTGGCTGAGACGAAAAACATATTCTCAATAAACCCTTTAGGGAAATAGGCCAGGTTTTCACCGTAACACGCCACATCTTGCGAATATATGTGTAGAAACTGCCGGAAATCGTCGTGGTATTCACTCCAGAGCGATGAAAACGTTTCAGTTTGCTCATGGAAAACGGTGTAACAAGGGTGAACACTATCCCATATCACCAGCTCACCGTCTTTCATTGCCATACGAAATTCCGGATGAGCATTCATCAGGCGGGCAAGAATGTGAATAAAGGCCGGATAAAACTTGTGCTTATTTTTCTTTACGGTCTTTAAAAAGGCCGTAATATCCAGCTGAACGGTCTGGTTATAGGTACATTGAGCAACTGACTGAAATGCCTCAAAATGTTCTTTACGATGCCATTGGGATATATCAACGGTGGTATATCCAGTGATTTTTTTCTCCATTTTAGCTTCCTTAGCTCCTGAAAATCTCGATAACTCAAAAAATACGCCCGGTAGTGATCTTATTTCATTATGGTGAAAGTTGGAACCTCTTACGTGCCGATCAACGTCTCATTTTCGCCAAAAGTTGGCCCAGGGCTTCCCGGTATCAACAGGGACACCAGGATTTATTTATTCTGCGAAGTGATCTTCCGTCACAGGTATTTATTCGCGATAAGCTCATGGAGCGGCGTAACCGTCGCACAGGAAGGACAGAGAAAGCGCGGATCTGGGAAGTGACGGACAGAACGGTCAGGACCTGGATTGGGGAGGCGGTTGCCGCCGCTGCTGCTGACGGTGTGACGTTCTCTGTTCCGGTCACACCACATACGTTCCGCCATTCCTATGCGATGCACATGCTGTATGCCGGTATACCGCTGAAAGTTCTGCAAAGCCTGATGGGACATAAGTCCATCAGTTCAACGGAAGTCTACACGAAGGTTTTTGCGCTGGATGTGGCTGCCCGGCACCGGGTGCAGTTTGCGATGCCGGAGTCTGATGCGGTTGCGATGCTGAAACAATTATCCTGAGAATAAATGCCTTGGCCTTTATATGGAAATGTGGAACTGAGTGGATATGCTGTTTTTGTCTGTTAAACAGAGAAGCTGGCTGTTATCCACTGAGAAGCGAACGAAACAGTCGGGAAAATCTCCCATTATCGTAGAGATCCGCATTATTAATCTCAGGAGCCTGTGTAGCGTTTATAGGAAGTAGTGTTCTGTCATGATGCCTGCAAGCGGTAACGAAAACGATTTGAATATGCCTTCAGGAACAATAGAAATCTTCGTGCGGTGTTACGTTGAAGTGGAGCGGATTATGTCAGCAATGGACAGAACAACCTAATGAACACAGAACCATGATGTGGTCTGTCCTTTTACAGCCAGTAGTGCTCGCCGCAGTCGAGCGACAGGGCGAAGCCC**ACCATGATTACGCCAAGCTCGAAATTAACCCTCACTAAAGGGAACAAAAGCTGGTACCTAACAGGATTAGTGCAATTCGAGTTGAATCACTGGGAAAAACATTGTCTTCTTTTTTATATTATCATTTGCATTAGTGCTGCAGTCGTAGATACTTGTTGGTTGAAAGACATCAGCTGGGAGGGACTGGACTAGCGTTTGGTAAGGAGACATACCTGTTAACGTTGGTTGCAAAATTCCATTTCGCGATTTATGTTATCTGTAAATCCTGATTTGTCTGGAATTCTTGATACTTCCGTTTTTTTAGAGGCCAATGATTAGCATCGGCGATTCTCAAAATAGCATTTTCGACATGCGGTGCTGATTTCATAAACATAGACAACGCTTTTACATGTAAAAGTAACTTGCGGACTTGGAACAGTGCTCTGTTTTTGGTGTGAACGTAACTCAGCAATATTTCTGTGCTAGCAAGGTTTTTTATGATCGACCGAAGATCTCAAAACTCCGGGTCTTTCAACTGTCTGACTAGACCATGTTCGTAACGTCGGACAGCAGCTTTCGTTGTACTGGTAGAATTTCTACGTGCGAAGCACGTGTAGGCAGGTTGAACGACGATCCCTGCCGATGGATGGATTGGCACGCGGCGGAACGCTTTCGTGATCTACACCACCTGGATCTTCACATATCTTCGAAATCGAAAAATTAACCAAGTCGACGGTATCGATAATATTCTAGCTGAGGGTACCCATGGCCAAGTTGACCAGTGCCGTTCCGGTGCTCACCGCGCGCGACGTCGCCGGAGCGGTCGAGTTCTGGACCGACCGGCTCGGGTTCTCCCGGGACTTCGTGGAGGACGACTTCGCCGGTGTGGTCCGGGACGACGTGACCCTGTTCATCAGCGCGGTCCAGGACCAGGTGGTGCCGGACAACACCCTGGCCTGGGTGTGGGTGCGCGGCCTGGACGAGCTGTACGCCGAGTGGTCGGAGGTCGTGTCCACGAACTTCCGGGACGCCTCCGGGCCGGCCATGACCGAGATCGGCGAGCAGCCGTGGGGGCGGGAGTTCGCCCTGCGCGACCCGGCCGGCAACTGCGTGCACTTCGTGGCCGAGGAGCAGGACTGACCGACGCCGACCAACACCGCCGGTCCGACGCGGCCCGACGGGTCCGAGGCCTCGGAGATCTGGGCCCATGCGGCCGCAACAACTACCTCGACTTTGGCTGGGACACTTTCAGTGAGGACAAGAAGCTTCAGAAGCGTGCTATCGAACTCAACCAGGGACGTGCGGCACAAATGGGCATCCTTGCTCTCATGGTGCACGAACAGTTGGGAGTCTCTATCCTTCCTTAAAAATTTAATTTTCATTAGTTGCAGTCACTCCGCTTTGGTTTCACAGTCAGGAATAACACTAGCTCGTCTTCACATCTTCCGCTGCATAACCCTGCTTCGGGGTCATTATAGCGATTTTTTCGGTATATCCATCCTTTTTCGCACGATATACAGGATTTTGCCAAAGGGTTCGCGTAGACTTTCCTTGGTGTATCCAACGGCGTCAGCCGGGCAGGATAGGTGAAGTAGGCCCACCCGCGAGCGGGTGTTCCTTCTTCACTGTCCCTTATTCGCACCTGGCGGTGCTCAACGGGAATCCTGCTCTGCGAGGCTGGCCGGCTACCGCCGGCGTAACAGATGAGGGCAAGCGGATGGCTGATGAAACCAAGCCAACCAGGAAGGGCAGCCCACCTATCAAGGTGTACTGCCTTCCAGACGAACGAAGAGCGATTGAGGAAAAGGCGGCGGCGGCCGGCATGAGCCTGTCGGCCTACCTGCTGGCCGTCGGCCAGGGCTACAAAATCACGGGCGTCGTGGACTATGAGCACGTCCGCGAGCTGGCCCGCATCAATGGCGACCTGGGCCGCCTGGGCGGCCTGCTGAAACTCTGGCTCACCGACGACCCGCGCACGGCGCGGTTCGGTGATGCCACGATCCTCGCCCTGCTGGCGAAGATCGAAGAGAAGCAGGACGAGCTTGGCAAGGTCATGATGGGCGTGGTCCGCCCGAGGGCAGAGCCATGACTTTTTTCACGTAACGGATCGGGGTGCGCGTGATTCGGAAGCACGTTCCATGGCCTCCATCAAGAAGAGGCACTTCGAGCTGTAAGTACATCACCGACGAGCAAGGCAAGACGATCCGTAACTATAACGGTCCTAAGGTAGCGAACTCGAG

**Sequence for URA3 cassette used for insertion into 25-3 and 26-3:**

GCATCGACATCAAGGATTTCTATCTTGGGACACCCATGGCTCGCTACGAATACATGCGCATCCCCGTCCCCGATATTCCACCAACCATTTTGGCTCAGTACCAGTTAGCTCCGCTCATTCACAACAACTCTGTTACCGTCGAAATTCGCAAAGGTATGTACGGCCTTCCCCAGGCCGGCATTCTAGCTCATGACCGCCTAGTTGAACACCTTGCTTGCCACGGCTACGTCAAAACCAAGCATACCGCCGGCCTTTTTCGACACGTCACACGCCCGATTCAATTTACCCTAGTTGTCGACAACTTTGGCATAAAATACACCGGCACCGAAAA

**Sequence for KanR-ARS cassette (ARS region shown in lower case:**

GCCAGTGTTACAACCAATTAACCAATTCTGATTAGAAAAACTCATCGAGCATCAAATGAAACTGCAATTTATTCATATCAGGATTATCAATACCATATTTTTGAAAAAGCCGTTTCTGTAATGAAGGAGAAAACTCACCGAGGCAGTTCCATAGGATGGCAAGATCCTGGTATCGGTCTGCGATTCCGACTCGTCCAACATCAATACAACCTATTAATTTCCCCTCGTCAAAAATAAGGTTATCAAGTGAGAAATCACCATGAGTGACGACTGAATCCGGTGAGAATGGCAAAAGCTTATGCATTTCTTTCCAGACTTGTTCAACAGGCCAGCCATTACGCTCGTCATCAAAATCACTCGCATCAACCAAACCGTTATTCATTCGTGATTGCGCCTGAGCGAGACGAAATACGCGATCGCTGTTAAAAGGACAATTACAAACAGGAATCGAATGCAACCGGCGCAGGAACACTGCCAGCGCATCAACAATATTTTCACCTGAATCAGGATATTCTTCTAATACCTGGAATGCTGTTTTCCCGGGGATCGCAGTGGTGAGTAACCATGCATCATCAGGAGTACGGATAAAATGCTTGATGGTCGGAAGAGGCATAAATTCCGTCAGCCAGTTTAGTCTGACCATCTCATCTGTAACATCATTGGCAACGCTACCTTTGCCATGTTTCAGAAACAACTCTGGCGCATCGGGCTTCCCATACAATCGATAGATTGTCGCACCTGATTGCCCGACATTATCGCGAGCCCATTTATACCCATATAAATCAGCATCCATGTTGGAATTTAATCGCGGCCTCGAGCAAGACGTTTCCCGTTGAATATGGCTCATAACACCCCTTGTATTACTGTTTATGTAAGCAGACAGTTTTATTGTTCATGATGATATATTTTTATCTTGTGCAATGTAACATCAGAGATTTTGAGACACAACGTGGCTTTGTTGAATAAATCGgatcgccaacaaatactaccttttatcttgctcttcctgctctcaggtattaatgccgaattgtttcatcttgtctgtgtagaagaccacacacgaaaatcctgtgattttacattttacttatcgttaatcgaatgtatatctatttaatctgcttttcttgtctaataaatatatatgtaaagtacgctttttgttgaaattttttaaacctttgtttatttttttttcttcattccgtaactcttctaccttctttatttactttctaaaatccaaatacaaaacataaaaataaataaacacagagtaaattcccaaattattccatcattaaaagatacgaggcgcgtgtaagttacaggcaagcgatcc
